# Virtual, Augmented, Mixed, and Immersive Technologies for Prenatal and Childbirth Education: Scoping Review

**DOI:** 10.2196/83621

**Published:** 2026-05-01

**Authors:** Susanna Pardini, Olga Navarro Martínez, Oscar Mayora Ibarra

**Affiliations:** 1 Digital Health Research Unit, Centre for Health and Wellbeing, Fondazione Bruno Kessler, Via Sommarive, 18, Trento, 38123, Italy; 2 Nursing Education and Care Research Group (GRIECE), Nursing Department, Faculty of Nursing and Podiatry, Universitat de València, Menéndez y Pelayo, Valencia, Spain

**Keywords:** extended reality, immersive technology, augmented reality, mixed reality, 360° video, virtual reality, childbirth education, parental education, prenatal education, breastfeeding

## Abstract

**Background:**

Virtual, augmented, mixed, and other immersive technologies, collectively referred to as extended reality (XR), are increasingly used to enhance experiential learning in health education. By creating interactive 3-dimensional or 360° environments, these technologies allow expectant parents to engage in realistic prenatal and childbirth scenarios, promoting emotional preparedness, knowledge acquisition, and confidence. Although XR has been widely studied in clinical training, its application in prenatal and childbirth education for parents remains less systematically explored.

**Objective:**

This scoping review aims to map and synthesize the current evidence on the use of virtual, augmented, mixed, and immersive technologies in prenatal and childbirth education, highlighting their educational benefits, methodological approaches, and implementation challenges.

**Methods:**

A comprehensive search was conducted across Scopus, Web of Science, PubMed, CINAHL, IEEE Xplore, APA PsycINFO, and APA PsycArticles from inception to October 16, 2025. Search terms included “virtual reality,” “augmented reality,” “mixed reality,” “extended reality,” and “immersive technology,” combined with prenatal and childbirth education descriptors. Studies were included if they applied immersive or XR technologies to deliver prenatal or childbirth education for expectant parents. Screening and data extraction were performed independently by 2 reviewers following PRISMA-ScR (Preferred Reporting Items for Systematic Reviews and Meta-Analyses extension for Scoping Reviews) guidelines. The review was registered on Open Science Framework (OSF).

**Results:**

From 1861 records, 11 studies from 8 countries were included, spanning randomized controlled, quasi-experimental, feasibility, and qualitative designs. Interventions comprised head-mounted display–based virtual reality, 360° video, and mixed reality simulations. Outcomes covered psychological, physiological, educational, and experiential domains. Most studies reported feasibility and high engagement, with encouraging signals for reduced anxiety and improved birth preparedness and, in some cases, reductions in pain and intrapartum indicators. No serious adverse events were reported; nausea and discomfort were infrequent and transient. Thematic analysis identified 5 recurring themes: enhanced birth preparedness, realism and presence as a key mechanism, usability barriers and the need for guided facilitation, motivational and educational potential, and limited partner inclusion. Methodological quality was heterogeneous, with small samples, nonstandardized measures, and short follow-up.

**Conclusions:**

Evidence for XR in prenatal education is promising yet preliminary. Rigorous multicenter studies with standardized outcomes, longer follow-up, and greater partner involvement, alongside attention to equitable access and digital literacy, are needed to support integration into maternity care pathways.

## Introduction

### Background and Importance of Prenatal Education

Prenatal education plays a pivotal role in preparing expectant parents for childbirth and early parenthood. Traditionally, such education has been delivered through in-person classes, with the goal of fostering confidence and informed decision-making during pregnancy [1-3]. Improving parental emotional readiness has also been associated with better neonatal outcomes, including reduced complications at birth, underscoring the indirect benefits of prenatal education on child health [4,5]. Beyond knowledge acquisition, prenatal programs aim to enhance emotional preparedness, self-efficacy, and engagement, factors that can significantly influence maternal and neonatal well-being [6].

### Limitations of Traditional Approaches

Despite its recognized importance, participation in conventional prenatal education programs may be complicated and at times impeded due to time constraints, work demands, and logistical or transportation barriers [7]. Traditional didactic formats often provide limited experiential engagement and may fail to adequately address psychological aspects such as fear of childbirth (FOC) and anxiety; a randomized controlled trial (RCT) evaluates an 8-week integrated childbirth education program that combines labor simulations with mindfulness practices to better target these needs. Compared with standard prenatal care, the program significantly reduced FOC, anxiety, and depressive symptoms and increased dispositional mindfulness through early postpartum, suggesting that integrative approaches (such as mindfulness) can improve the impact of the traditional prenatal education programs, for example, improving perinatal mental health in women with high FOC [8]. Pregnancy is widely recognized as a sensitive period in which psychological distress, including fear, anxiety, and depression, can negatively affect both maternal and neonatal outcomes [5,9]. Common concerns include miscarriage, fetal abnormalities, labor pain, and the perceived ability to be a “good mother.” Studies have shown that FOC, particularly in late pregnancy, may contribute to avoidance behaviors such as elective cesarean section [9,10]. Nonpharmacological interventions, including hypnosis, physical activity, and cognitive-behavioral strategies, have been trialed to mitigate childbirth-related anxiety with mixed but promising results [11-13]. Overall, prenatal education remains a widely accessible and effective strategy to empower parents and promote positive maternal and infant outcomes [14-16].

### The Use of Virtual, Augmented, Mixed, and Immersive Technologies in Prenatal Education

With the growing digitization of health care and the evolving needs of diverse parent populations, innovative approaches to prenatal education are increasingly being explored.

Extended reality (XR) is an umbrella term encompassing virtual reality (VR), augmented reality (AR), and mixed reality (MR) technologies, which collectively enable users to interact with digital environments and objects beyond traditional screen-based media. XR systems differ in the degree of immersion and integration between virtual and physical elements, including (1) VR, which provides full immersion in a computer-generated environment, typically via a head-mounted display (HMD) that occludes the physical world; (2) AR, which overlays digital information or objects onto the real environment, maintaining users’ awareness of their surroundings; and (3) MR, which allows real and virtual elements to coexist and interact in real time, creating a seamless hybrid environment. These immersive systems leverage high-resolution audiovisual displays, motion tracking, and multisensory feedback (eg, haptic or spatial audio) to generate a sense of presence and involvement, key dimensions of the immersive experience [17,18]. In this review, XR and immersive technologies are defined as digital simulation environments, ranging from fully immersive VR headsets to semi-immersive 360° video and mobile-based apps, designed to support experiential learning and emotional engagement during prenatal and childbirth education [19,20]. By providing immersive, interactive simulations, XR could enable users to experience realistic childbirth environments in a safe and controlled setting, potentially enhancing understanding, emotional preparedness, and engagement, especially among first-time parents or those with limited access to conventional resources [21]. While the use of XR in clinical training for health care professionals is well established [22], its application for patient-facing prenatal education remains relatively understudied. Early studies suggest that immersive interventions, administered in different phases of the childbirth process, may reduce childbirth-related anxiety, improve systolic blood pressure, decrease maternal heart rate, and even increase fetal movement acceleration [23,24]. Furthermore, XR-based interventions can offer increased flexibility and accessibility, potentially reducing geographic and socioeconomic barriers to participation [25].

Recent randomized trials and systematic reviews have begun to consolidate the evidence base for immersive technologies in perinatal care. VR used during labor or cesarean birth has been associated with reductions in pain and anxiety and improvements in birth satisfaction, without major safety concerns for mothers or newborns [21,26,27]. In parallel, VR-based prenatal education and broader programs for pregnant women have reported gains in childbirth knowledge, self-efficacy, and pregnancy-related anxiety, suggesting that immersive tools can strengthen both cognitive and emotional preparation for birth [24]. These developments highlight the need for a focused synthesis of XR specifically in parent-facing prenatal and childbirth education, beyond the more established literature on clinician training.

### Public Health and Psychosocial Context

Beyond technological innovation, prenatal education is a key component of global maternal health policy. The World Health Organization (WHO) has emphasized the importance of high-quality, integrated maternity services that ensure a positive pregnancy and childbirth experience [28]. Within this framework, addressing maternal emotional well-being, particularly anxiety and FOC, is considered a central priority. Immersive technologies can contribute to these goals by providing emotionally engaging and accessible educational experiences, supporting both knowledge acquisition and psychological resilience during pregnancy [29,30].

### Pedagogical Frameworks Supporting the Use of Immersive Technologies

The educational value of immersive technologies is supported by several pedagogical models. The Interaction Model of Client Health Behavior (IMCHB) emphasizes the role of emotionally resonant and individualized learning experiences in shaping behavior change [31]. Likewise, constructivist learning theory posits that experiential and interactive simulations facilitate understanding through active, context-based engagement, allowing learners to integrate new information with prior experiences [32]. The Cognitive Load Theory offers a complementary framework, suggesting that well-designed XR content can enhance learning by minimizing extraneous cognitive demands and improving retention through multimodal cues such as narration, interactivity, and visual emphasis [33]. Together, these models support the potential of XR-based prenatal education to foster active, meaningful, and self-directed learning.

### Challenges and Research Gap

Despite its promise, XR-based prenatal education faces several challenges, including usability issues (eg, cybersickness, device discomfort, and limited digital literacy), ethical concerns about replacing human interaction, and potential inequalities in access. Existing research is also limited by methodological heterogeneity, small sample sizes, and a lack of longitudinal follow-up, making it difficult to generalize findings or establish best practices (eg, [34,35]). As prenatal care becomes increasingly hybrid and digitally mediated, particularly after the COVID-19 pandemic, it is essential to understand how, when, and for whom XR interventions are most effective, and to identify key design and implementation features.

### Aim of the Review

This scoping review aims to explore and map the evidence on the application of immersive technologies in prenatal course programs, focusing on their benefits, challenges, and potential for enhancing learning and emotional preparedness among expectant parents. By synthesizing key findings and trends, the review aims to inform educators, clinicians, designers, policymakers, and researchers. It supports evidence-informed use of immersive technologies to improve maternal and perinatal outcomes and fosters a shared understanding of the state of the art and next research steps in partnership with stakeholders involved in childbirth preparation. This scoping review begins with a comprehensive search of academic peer-reviewed literature. By grounding the review in scientifically validated evidence, it lays a basis for later examining broader practical implications and innovations within the gray literature. The following key research questions (RQs) guided the search strategy and data extraction process in the academic literature:

RQ1: what are the primary applications of virtual, augmented, mixed, and immersive technologies in prenatal education programs?
RQ2: what benefits have been reported regarding their use in enhancing learning, emotional preparedness, and engagement?
RQ3: what challenges and limitations are identified in implementing XR-based interventions in prenatal education?
RQ4: what methodologies and technological approaches are most used in studies on XR in prenatal education?
RQ5: what research gaps exist in this area, and what future directions are recommended?

The findings will provide a thorough overview of how XR is used in prenatal education, offering insights for researchers, educators, and policymakers on effectively integrating innovative technologies to enhance maternal health outcomes.

## Methods

### Study Design

This scoping review followed a five-step process: (1) formulating the research question, (2) identifying relevant studies, (3) selecting studies for inclusion, (4) organizing the data, and (5) synthesizing and presenting the findings [36]. The final search strategy was refined through an iterative approach to optimize database searches and improve search terms. To ensure methodological rigor and clarity, the study design and manuscript preparation adhered to the PRISMA-ScR (Preferred Reporting Items for Systematic Reviews and Meta-Analyses extension for Scoping Reviews) framework [36,37].

A scoping review was chosen to provide an overview of the available evidence, not a formal critical synthesis or a definitive answer to a specific research question [38].

### Protocol and Registration

The scoping review protocol is registered a priori with the Center for Open Science (OSF; registration type: OSF Preregistration, date registered: March 5, 2025 [39]).

### Selection of Sources of Evidence and Eligibility Criteria

The selection of evidence sources was conducted in 4 sequential and distinct phases: “First Round,” “Second Round,” “References Inclusion Round,” and “Third Round” (Table 1). To systematically identify a comprehensive set of papers that contribute to addressing our research questions, we established a set of eligibility criteria to be applied iteratively through several rounds. The criteria aim to minimize false negatives and false positives, ensuring that only studies meeting the defined standards are included in the review. Table 1 provides an overview of the criteria used to filter and select the studies.

**Table 1.** Description of the eligibility criteria.

Selection sources phase	Eligibility criteria applied
First round	Inclusion criteria Original papers published in English. Articles published in peer-reviewed journals or credible conference proceedings. Focused on the use of XR^a^ technologies (VR^b^, AR^c^, MR^d^, or 360° video) in prenatal or childbirth education for pregnant women and expectant parents. Addressed outcomes such as learning, emotional preparedness, anxiety reduction, or partner engagement. Involved expectant parents and/or stakeholders. Exclusion criteria Non–English-language Article types: book, book chapters, dissertations, abstracts, posters, interviews, protocols, and reports. Nonoriginal articles: meta-analysis and reviews, including reviews without original data or studies with insufficient detail. No full text available. Not focused on prenatal or childbirth education for parents. Addressed XR in clinical obstetric care but not in an educational context. Studies primarily describing XR-based training for health care professionals or clinical skills education. Studies based solely on remote learning, online courses, or mobile messaging without immersive interaction.
Second round	Exclusion criteria Studies without participants. The article does not report any qualitative or quantitative participant involvement results.
References inclusion round	This step focused on identifying additional studies by screening the reference lists of the previously included papers.
Third round	This phase consisted of an additional verification of all previously screened files to ensure consistency and completeness. Furthermore, we extended the search to include the SciSpace database, which allowed us to identify and incorporate additional relevant papers not captured in earlier rounds.

a XR: extended reality.

b VR: virtual reality.

c AR: augmented reality.

d MR: mixed reality.

### Information Sources and Search Strategy

A comprehensive search strategy was developed to identify relevant literature on the use of XR in prenatal education. The search process included both database searches and gray literature exploration to ensure thorough coverage of the topic.

The search was conducted from January 30 to October 16, 2025. To ensure comprehensive coverage across health sciences, psychology, and technology, the following databases were systematically searched:

1. APA PsycArticles and APA PsycINFO: capture psychological and behavioral science research, including mental health, anxiety, and parental preparedness relevant to prenatal education.
2. PubMed: offers broad biomedical coverage, including maternal health, obstetrics, and digital health, making it critical for identifying studies on physiological and emotional outcomes.
3. Web of Science (WoS), Cumulative Index to Nursing and Allied Health Literature (CINAHL)**,** and Scopus**:** provide wide-reaching coverage of peer-reviewed, multidisciplinary research, ensuring inclusion of various study designs (eg, RCTs and pilot studies).
4. IEEE Xplore: specializes in technology and human-computer interaction (HCI), essential for identifying studies on VR systems, usability, and digital learning tools.

The gray literature was explored using the following sources: Google Scholar**,** WHO Reproductive Health Library, and SciSpace.

The search strategy combined three main concept blocks: (1) immersive technologies (“virtual reality,” “augmented reality,” “mixed reality,” “extended reality,” and “immersive technology”); (2) prenatal and childbirth education (“prenatal education,” “antenatal education,” “childbirth education,” “prenatal classes,” “parenting classes,” “expectant parent education,” and “childbirth training”); and (3) participant and facilitator roles (“pregnant women,” “expectant parents,” “midwife,” “childbirth educator,” “parent educator,” “perinatal educator,” “healthcare provider,” and “health professional”).

**Figure 1.**
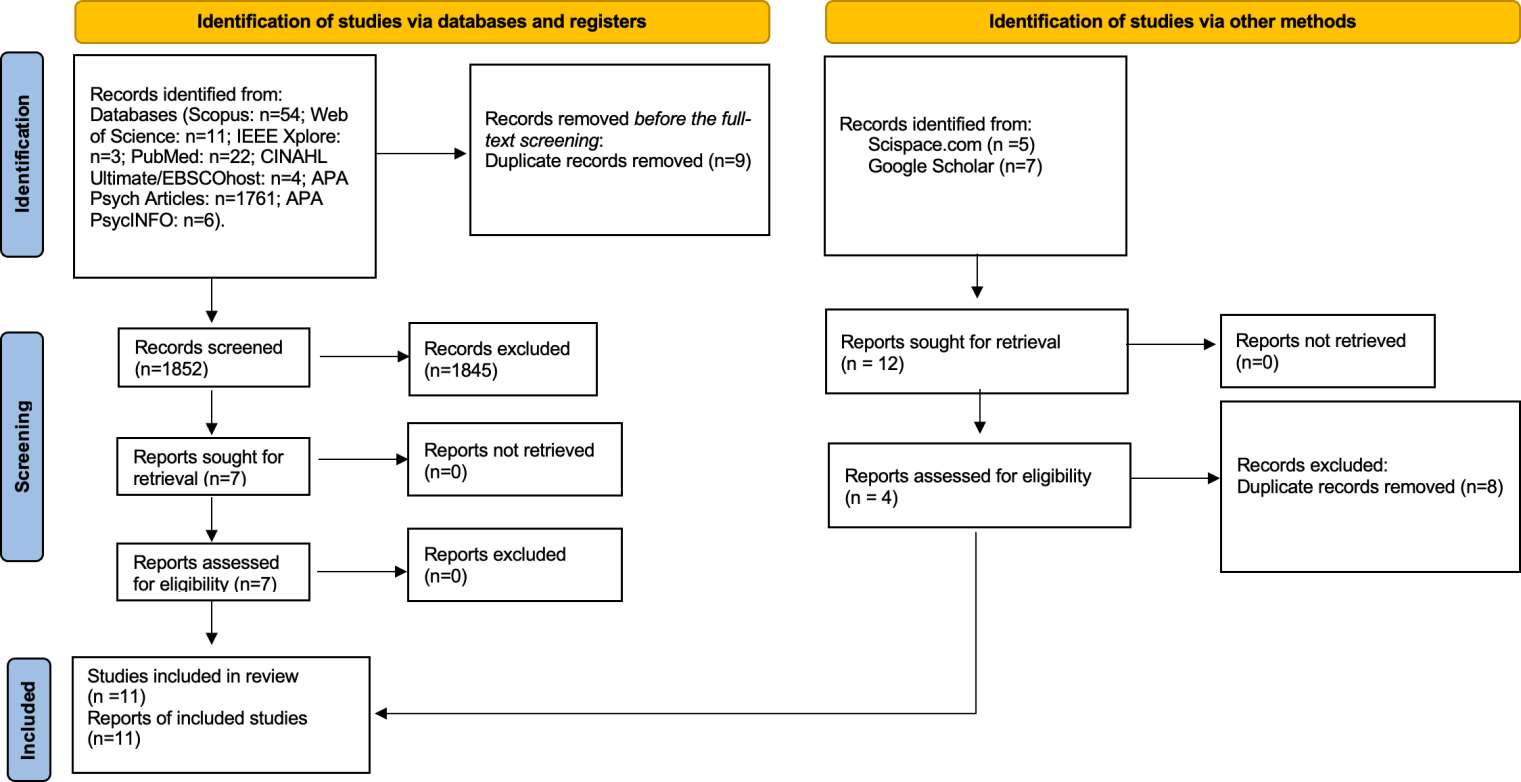
PRISMA (Preferred Reporting Items for Systematic Reviews and Meta-Analyses) 2020 flow diagram for new systematic reviews which included searches of databases, registers, and other sources [51]. Adapted from Page et al [51], which is published under a Creative Commons Attribution 4.0 International License (CC BY 4.0 [52]).

Boolean operators (“AND”/“OR”/“NOT”) were applied appropriately, and syntax was adapted to each database (PubMed, Scopus, Web of Science, IEEE Xplore, CINAHL Ultimate, APA PsycINFO, and APA PsycArticles). The final search was rerun on October 16, 2025, and full database-specific queries are reported in Multimedia Appendix 1. Studies focusing exclusively on professional training, without a direct patient-facing component, were excluded. This approach balances inclusivity in the initial search with relevance during study selection, helping to identify interventions co-designed or delivered by professionals but intended for parents. The search strategy, including the keywords and search strings used, is summarized in Table 1. The research team supplemented the sources identification process by including references cited within the full texts of publications included as a result of the database search. This approach was guided by the selection strategy detailed in the PRISMA (Preferred Reporting Items for Systematic Reviews and Meta-Analyses) 2020 flow diagram (Figure 1).

### Data Items

For each included study, we extracted the following variables: (1) bibliographic information: authors, year of publication, country, and journal or conference; (2) study characteristics: design (eg, RCT, quasi-experimental, and qualitative), setting (hospital, university, and online), and sample size; (3) participant characteristics: demographics (age, sex, and gestational stage), inclusion of partners or caregivers, and specific eligibility criteria; (4) intervention details: type of VR technology (eg, immersive headset, 360° video, and mobile app), educational content (childbirth, breastfeeding, prenatal exercise, and mental health), frequency, and duration of sessions; (5) comparator (if applicable): conventional prenatal education, standard care, or no intervention; (6) outcomes: (a) psychological features and symptoms (anxiety, depression, fear of childbirth, and perceived stress); (b) physiological during intrapartum and postpartum outcomes (pain, uterine activity, perineal trauma, and postpartum hemorrhage); (c) educational outcomes (knowledge acquisition, memory retention, and preparedness); and (d) user experience outcomes: usability, satisfaction, immersion, and engagement; (6) main results: direction of outcomes, qualitative themes, and feasibility and acceptability measures; and (7) other relevant information: inclusion of partner or family support, equity considerations (digital literacy and access), and reported methodological limitations (Tables 2-6).

**Table 2.** General information summaries of selected studies*.*

References	Database	Country	Study design	Background	Objectives
Xie and Zeng [23]	PubMed	China	Randomized clinical trial with a 2-group intervention design	With the implementation of the 2-child policy in China, childbirth rates have increased. Many women experience fear and anxiety about childbirth, which can prolong labor and increase complications. VR^a^ has been increasingly used in health care settings, offering immersive and interactive experiences. This study investigates the effectiveness of VR combined with moderate perineal protection during childbirth.	To explore the effects of VR-based prenatal health education combined with moderate perineal protection on childbirth outcomes, pain management, self-efficacy, anxiety, and perineal trauma in primiparous women.
Lee et al [34]	Google Scholar	Korea	Prospective interventional study	Pregnant women face significant physiological and psychological changes, increasing the risk of mental health disorders, including depression and anxiety. Mental health disorders in pregnancy can negatively impact both the mother and infant, increasing risks such as low birth weight and preterm birth. Existing mental health programs for pregnant women are insufficient in terms of accessibility and effectiveness.	To prospectively investigate the efficacy of a VR-assisted mental health promotion program in pregnant women. To assess its impact on depression, anxiety, perceived stress, and quality of life. To evaluate the program’s effectiveness in high-risk subgroups of pregnant women.
Caballero-Galilea et al [40]	Web of Science, Scopus, and PubMed	Spain	Single-group pre-post quasi-experimental study based on the Transparent Reporting of Evaluations with Nonrandomized Designs (TREND) guidelines	Anxiety affects up to 1 in 4 pregnant women and is associated with adverse obstetric, neonatal, and psychological outcomes. FOC^b^ occurs in 5%‐15% of cases and contributes to higher cesarean rates and reduced maternal well-being. While traditional prenatal education and psychological interventions can mitigate these effects, evidence on the use of VR for improving mental health and reducing childbirth-related anxiety remains limited but promising.	To assess the effectiveness of immersive VR exposure to the childbirth process in reducing anxiety among women in the third trimester of pregnancy.
Siivola et al [41]	CINAHL Ultimate, PubMed, and Google Scholar	Finland	Pilot study with a user-centered design process	The COVID-19 pandemic led to the cancellation or transition of CBE^c^ to online formats in Finland. There was a need for an alternative method to provide immersive and engaging CBE. FOC has been increasing in Finland, necessitating better education tools.	To develop a VR childbirth education program with 360° videos. To test the program’s usability, motion sickness, effectiveness, and potential impact on FOC. To investigate which end-user devices are preferred (eg, VR headset, computer, tablet, and smartphone). To make CBE accessible across multiple devices, including VR headsets, computers, tablets, and smartphones.
Siivola et al [42]	Scopus and Google Scholar	Finland	User-centered design study	CBE in Finland has deteriorated, with many expectant parents lacking access to hospital tours or comprehensive prenatal education. VR presents an opportunity to enhance CBE by offering immersive and realistic childbirth experiences.	To evaluate the effectiveness of a VR CBE program in improving learning outcomes, usability, and user experience, while also assessing its impact on FOC.
Tang et al [43]	IEEE Xplore	Belgium	Qualitative user study	Breastfeeding has well-documented health benefits, yet many new parents face difficulties during the early postnatal stage. Traditional antenatal education often fails to convey the complexity of real-life breastfeeding experiences. VR has the potential to provide an immersive and interactive way for parents-to-be to explore these challenges.	To assess the potential of a VR breastfeeding simulation (“Virtual Feed”) in conveying the lived experience of breastfeeding and to explore user expectations related to playful technology in health care.
Tang et al [44]	Scopus, Web of Science, and Google Scholar	Belgium (participants from various backgrounds including Western, Eastern, and Southern Europe, as well as South and Southeast Asia)	Qualitative co-design	Breastfeeding offers numerous benefits but remains challenging for many parents. Antenatal education often presents breastfeeding as a natural and straightforward process, which can lead to unrealistic expectations. The study aimed to address this gap by designing an immersive VR breastfeeding simulation to help parents experience common breastfeeding challenges.	The study sought to answer 2 primary research questions: What design considerations are needed to create interactive simulations for breastfeeding experiences? How do parents and parents-to-be perceive such simulations, and what is their potential in antenatal education?
Noben et al [45]	Google Scholar	Netherlands	Randomized controlled trial	Previous research demonstrated that the level of anxiety and FOC is known to be associated with the incidence of postpartum depression. Women who deliver by a CD^d^ are at risk for both increased fear of childbirth and postpartum depression. It is essential to minimize preoperative anxiety for these patients because lower preoperative anxiety has been shown to lead to greater maternal satisfaction with CD and thus a more positive birth experience.	To investigate the effect of VR in addition to conventional information provision on the preoperative anxiety levels of women undergoing a planned CD.
Setiawan et al [46]	Google Scholar	Indonesia	Pilot feasibility study; Semiexperimental study	There are still many pregnant women who are less interested in exercising during pregnancy, due to heavy workload during the working day and a dense schedule of daily activities in her career or as a housewife. Some women assume that attending a pregnancy exercise course in hospitals or health care centers is time-consuming and too formal because they have to follow the prenatal personal trainer schedule. The technology that allows helping pregnant women in exercise during pregnancy is virtual reality.	To develop a VR app for pregnant women to easily perform an exercise during pregnancy with no limited space and time.
Park and Kim [47]	Google Scholar	Korea	Quasi-experimental study	Hospitalized women with threatened preterm labor often experience psychological and physical distress, which may exacerbate uterine activity and cervical changes, necessitating targeted prenatal care. VR-based prenatal education may improve engagement and support. Guided by the Cox IMCHB^e^, this study developed and evaluated a VR prenatal education program to address these complex needs.	To design, administer, and assess the impact of a VR-based prenatal education program for pregnant women hospitalized due to preterm labor.
Montoya-Moncada et al [48]	PubMed	Colombia	Noninferiority randomized controlled trial	Breastfeeding is natural but requires learned skills and is shaped by social and psychological factors. The World Health Organization and the United Nations Children’s Fund recommend 6 months of exclusive breastfeeding and continued breastfeeding with complementary foods for at least 2 years, yet rates remain low worldwide. Traditional counseling is mostly theoretical and rarely uses modern technology; motivation and confidence help success, while stigma, aggressive formula marketing, and poor guidance hinder it. This study tests whether a mixed-reality educational approach, compared with standard counseling, improves mothers’ satisfaction and confidence.	To test whether mixed reality + standard counseling improves maternal self-efficacy (BSES-SF) and satisfaction (MBFES) versus standard counseling alone, 1 week post partum.

a VR: virtual reality.

b FOC: fear of childbirth.

c CBE: childbirth education.

d CD: cesarean delivery.

e IMCHB: Interaction Model of Client Health Behavior.

**Table 3.** Demographic characteristics of participants in the experiments*.*

Study (year)	Single or multicenter	Inclusion and exclusion criteria	Recruitment methods	Sample size	Sex, n (%)	Age (years)^a^
Xie and Zeng [23] (2023)	Single center	Inclusion criteria: (1) singleton full-term pregnancy, (2) normal external pelvic measurement, (3) no obvious cephalopelvic disproportion, (4) no complications during pregnancy or labor, and (5) age 20‐34 years. Exclusion criteria: (1) estimated fetal weight≥4000 g, (2) fetal biparietal diameter>9.7 cm, (3) presence of mental illness or communication disorders, and (4) use of analgesia during labor.	Pregnant women receiving routine antenatal care at the hospital between June 1, 2018 and December 31, 2018, were invited to participate. Participants and their families provided informed consent before enrollment.	N=200: primiparous women were randomly assigned to either the treatment group (n=100) or the control group (n=100).	Female=200 (100%)	Treatment group: 30.29 (1.14) Control group: 30.35 (1.07)
Lee et al [34] (2025)	Single center	Inclusion criteria: (1) pregnant women, (2) able to understand and use the Korean language, and (3) willing to participate in the study. Exclusion criteria: (1) intellectual disability preventing VR^b^ training, (2) visual impairment making VR use impossible, and (3) illiteracy.	Advertisements in the obstetrics and gynecology unit.	N=56 (participants were grouped using cluster analysis based on their mental health characteristics before the VR intervention. The study identified three clusters: (1) Cluster 1 (Healthy Mental State): 19 (33.9%) participants with low depression and anxiety. Higher quality of life. No significant need for intervention. Cluster 2 (Treatment-Required Group—High-Risk): 9 (16.1%) participants with high depression and anxiety scores. Low quality of life. Required active mental health support. Cluster 3 (Intermediate Group): 28 (50%) participants with moderate depression and anxiety. NB: High-risk participants were also defined using standard clinical cutoff scores: (1) PHQ-9^c^ cutoff for depression:≥8; (2) GAD-7^d^ cutoff for anxiety:≥5. From this, two additional high-risk groups were analyzed separately: (1) high-risk for depression (PHQ-9≥8); n=20 (35.7%); (2) high-risk for anxiety (GAD-7≥5); n=8 (14.3%).	Female=56 (100%).	34.3 (4.0)
Caballero-Galilea et al [40] (2025)	Single center	Inclusion criteria: (1) aged 18 years or older, (2) nonmultiple pregnancy, and (3) be either primiparous or multiparous in the third trimester of gestation. Exclusion criteria: (1) women with visual or hearing impairments that prevent effective participation in virtual reality experiences, (2) those with neurological, otorhinolaryngological, or mental health conditions that could be exacerbated using VR devices.	Pregnant women attending a prenatal education program in a health center in Madrid (Spain) between February 2023 and October 2024.	N=73 pregnant women.	Female=73 (100%).	Median 34.0 (IQR 5.0)
Siivola et al [41] (2023)	Single center	Inclusion criteria: (1) women who had recently given birth but were not currently pregnant. Exclusion criteria: (1) pregnant women (for the initial pilot), (2) individuals with a prior diagnosis of FOC^e^ (not included in the first phase).	Participants were recruited through an online survey and invited to test the program in a research facility. A second phase allowed participants to test the program remotely.	First phase: N=3 women who had recently given birth. Second phase: open to more participants, allowing home testing.	Female=3 (100%)	Not explicitly stated in the document.
Siivola et al [42] (2024)	Single center	Inclusion criteria: (1) pregnant individuals fluent in Finnish who had received CBE^f^ from public prenatal clinics but had not been offered a hospital tour. Exclusion criteria: (1) individuals not pregnant, non-Finnish speakers, or those unable to participate in the research location for initial testing.	Participants were recruited through social media.	N=5 pregnant women.	Female=5 (100%)	37 (SD not reported); 31‐41 years
Tang et al [43] (2022)	Single center	Inclusion criteria: (1) breastfeeding parents, parents-to-be, and partners interested in antenatal education. Exclusion criteria: (1) not explicitly mentioned but likely excluded those with significant VR interaction limitations or lack of consent.	Snowball sampling. Flyers at a local daycare, university campus, and social media outreach.	N=10: Breastfeeding parents (n=6) Parents-to-be (n=3) Partner (n=1)	Female=9 (90%) Male=1 (10%)	25‐45 years (n=8); 36‐45 years (n=2)
Tang et al [44] (2022)	Single center using online and in-person methodologies	Inclusion criteria: (1) participants included breastfeeding parents, partners, and parents-to-be who planned to breastfeed. Exclusion criteria: (1) no specific exclusion criteria mentioned apart from not having relevant breastfeeding experience or interest.	Participants were recruited through snowball sampling with advertisements on social media and word-of-mouth referrals.	N=19: Breastfeeding mothers (n=11), Partners (n=5), Parents-to-be who planned to breastfeed (n=3), and they had 1 child (n=11), 2 children (n=2), and 3 children (n=3). The youngest child was 15 months old (SD 9.45), and the breastfeeding duration of the youngest child was up to 1 month (n=1), up to 3 months (n=2), up to 6 months (n=2), up to 9 months (n=1), and up to one year or longer (n=10). Participants resided in Western Europe, but grew up in Western Europe (n=6), Eastern Europe (n=5), Southern Europe (n=4), South Asia (n=1), Southeast Asia (n=1), East Asia (n=1), South America (n=1).	Female=13 (68%) Male=6 (32%)	26‐45 years (n=13); 36-45 years (n=6)
Noben et al [45] (2019)	Single center	Inclusion criteria: women aged 18 years or older, had planned for elective CD^g^ after 37 weeks of gestation, and had sufficient knowledge of the Dutch language. Exclusion criteria: prematurity (gestational age<37 weeks), placenta previa, pre-eclampsia, and a suspected congenital anomaly.	Patients were recruited from the outpatient clinic at our hospital, enrolled from November 2016 to January 2018, (who were scheduled for elective CD at Máxima Medical Center in Veldhoven).	N=97: Virtual reality group=49 and control group=48 The partners also participated, but it is not indicated how many of them collaborated in the study.	Female=97 (100%)	VR group 32.6 (3.9); Control group 33.12 (4.3)
Setiawan et al [46] (2019)	Single center	(Inclusion and exclusion criteria are not labeled explicitly) Inclusion criteria: provide voluntary consent to engage with the VR system. Gestational age starts from the age of 16‐18 weeks.	(Not labeled explicitly) Convenience sampling ; voluntary participation ; prescreening for medical eligibility.	N=6 women	Female=6 (100%)	27.7 (2.3)
Park and Kim [47]	Multicenter study (2 hospitals of South Korea were involved. One hospital was assigned to conduct the experimental intervention, while the other was used to collect control group data)	Inclusion criteria: pregnant women hospitalized for preterm labor.	Convenience sampling.	N=31: n=15 (VR group); n=16 (control group).	Female=31 (100%)	VR group: 30.26 (3.63); Control group: 31.43 (2.50)
Montoya-Moncada et al [48] (2025)	Single center	Inclusion criteria: third-trimester pregnant women≥18 years, hemodynamically stable, oriented, and intending to breastfeed. Exclusion criteria: neonatal malformations or conditions affecting breastfeeding, and maternal contraindications (eg, HIV+, oncologic treatment), NICU^h^ or ICU^i^ need.	Eligible women attending prenatal care; consent obtained during prenatal sessions; intervention delivered within maternity and/or paternity preparation courses.	N=58 randomized (MR+ standard training group: n=29; control group: n=29); 76 screened; no loss to follow-up reported.	Female=58 (100%)	Not reported; eligibility≥18 years

a Age data are reported as mean (SD), median (IQR), range, or categories, as available.

b VR: virtual reality.

c PHQ-9: Patient Health Questionnaire-9.

d GAD-7: Generalized Anxiety Disorder-7.

e FOC: fear of childbirth.

f CBE: childbirth education.

g CD: cesarean delivery.

h NICU: neonatal intensive care unit.

i ICU: intensive care unit.

**Table 4.** Characteristics of experimental methodology.

Study	Experimental procedure	Health operator involvement	Measures	Variables investigated
Xie and Zeng [23]	Treatment group: prenatal education with VR^a^-based health education. Control group: traditional prenatal education.	Midwives trained in moderate perineal protection. Nursing staff provided VR-based health education.	Delivery outcomes: time of second-stage labor, postpartum hemorrhage within 2 hours, neonatal Apgar scores, and neonatal weight. Pain levels: VAS^b^ (0‐10). Anxiety levels: VAS-A. Self-efficacy: general Self-Efficacy Scale. Quality of life satisfaction: self-reported scale (0‐100).	Pain. Anxiety. Delivery outcomes (labor duration, hemorrhage, and perineal trauma). Self-efficacy and quality of life.
Lee et al [34]	Experimental procedure: VR-assisted mental health promotion program designed specifically for pregnant women.	Health care professionals were involved in the recruitment process and monitoring participant safety. VR procedure was self-administered Assessment and monitoring: the mental health state of participants was assessed before and after the VR intervention using self-reported measures.	Standardized scales and questionnaires: PHQ-9^c^ GAD-7^d^ PSS^e^ WHOQOL-BREF^f^	Depressive and anxiety symptoms. Stress-related symptoms Quality of life.
Caballero-Galilea et al [40]	Participants experienced a 60-min immersive VR simulation of eutocic childbirth after standard education. Anxiety (PRAQ-20^g^) was assessed pre- and postintervention.	Developed and supervised by midwives and obstetricians, midwives facilitated sessions and ensured participant safety during the VR simulation.	Pregnancy-Related Anxiety Questionnaire–Revised (PRAQ-20) administered pre and post VR; sociodemographic and obstetric data collected.	Pregnancy-related anxiety VR adverse effects.
Siivola et al [41]	Participants used VR headsets and other devices to test the childbirth education program.	The project involved midwives, childbirth educators, and researchers. A real midwife played the role in the videos to ensure authenticity.	Pretest Questionnaire: a structured online questionnaire was administered before participants engaged with the VR program. During the VR experience, observational measures, and a cognitive walkthrough were conducted to assess usability. After the VR experience: semistructured interview to gain qualitative feedback. Posttest Questionnaire: to evaluate their experiences. Usability and interaction tracking (during home use phase).	Usability of the VR program. Motion sickness occurrence. User preference for different devices. Impact on FOC^h^. Variables measured: Demographics: name, age, number of previous births, last childbirth year, current pregnancy status. Previous childbirth education exposure: participation in childbirth education (CBE) at a public prenatal clinic, attendance at a hospital birth ward tour, enrollment in additional childbirth education classes Preferred learning methods for childbirth. FOC history Experience with virtual reality. Engagement with the VR program. Motion sickness symptoms. Usability and learning curve. Time spent in the VR experience. Duration of headset use. Physical comfort. Device preference. Perceived value of VR components. Effectiveness of different media formats in learning. Impact on fear of childbirth.
Siivola et al [42]	Participants used the VR childbirth education program with a headset under researcher observation.	A Lamaze-certified childbirth educator was involved in study design, data analysis, and participant support.	Usability: PSUS^i^ Engagement: observational data on VR session length and content consumption Learning outcomes: self-reported understanding of childbirth-related topics. Emotional impact: FOC	User engagement and experience with VR childbirth education. Usability of the VR program Improvement in childbirth knowledge and preparedness. Impact on FOC.
Tang et al [43]	Participants engaged with the VirtualFeed VR breastfeeding simulation.	None reported; study focused on self-guided user experiences.	Qualitative thematic analysis of interview transcripts. User feedback on realism, effectiveness, and engagement with the simulation.	User perceptions of breastfeeding simulation realism. Impact of interactivity and game-like elements. Potential application of VR for antenatal education.
Tang et al [44]	Participants engaged in (1) visualizing and discussing breastfeeding settings; (2) exploring challenges and social influences on breastfeeding; (3) expressing design preferences for the VR environment; and (4) describing ideal and challenging breastfeeding scenarios.	The study did not involve direct medical professionals.	Thematic analysis of participant responses. Visual and interactive engagement through VR and design workshops. Reflections on breastfeeding experiences based on user feedback.	User perception of the breastfeeding simulation Immersion and realism of the VR experience Potential impact on antenatal education Challenges and facilitators of breastfeeding.
Noben et al [45]	VR group: VR video was shown using the Infor-Med app on the participant’s smartphone, and VR glasses were supplied by the researcher at the outpatient clinic.	Physician and researcher	Anxiety: VAS-A. Simulation Sickness: SSQ^j^. Tilburg Pregnancy Distress Scale. Childbirth Perception Scale (CPS). Pregnancy and Childbirth Questionnaire (PCQ).	Preoperative anxiety, motion sickness symptoms, woman’s perception of her pregnancy, perception of delivery and perception of the first postpartum week, preoperative information, participants in the VR group received an additional question if they felt more prepared for CD after seeing the VR video.
Setiawan et al [46]	Doing vital sign check-up to the target user before practice with VR. VR application implementation for the target user. Doing a vital sign check-up after practice with VR and evaluation of the IVE^k^ questionnaire.	None reported.	Simulator Sickness Questionnaire, User experience in the IVE.	Vital signs: heart rate (HR), blood pressure (BP), respiration rate (RR), and anemic conjunctiva (AC). Symptoms like vomiting, nausea, and dizziness.
Park and Kim [47]	VR Group: 3 VR sessions. Components: educational VR program and relaxation meditation VR. Control Group: received usual care only without VR.	1 VR expert, 1 nursing professor, and 1 head nurse from a women’s specialty hospital.	Self-report questionnaire: STAI^l^. Stress related to preterm labor. Pregnancy health care practice behavior. Self-efficacy in pregnancy health care. Uterine contractions: frequency (times/min) and intensity (mm Hg). Cervical length: transvaginal ultrasound.	State anxiety, stress related to preterm labor, frequency and intensity of uterine contractions, cervical length, practice behaviors in pregnancy health care, self-efficacy in health care.
Montoya-Moncada et al [48]	Intervention: a single facilitated session added to standard counseling, including a headset-based immersive video and hands-on practice using a silicone breast model, infant doll, and neonatal simulator. Control: standard counseling only.	Trained nurses, nursing assistants, and general practitioners delivered and facilitated sessions and provided real-time feedback.	Primary: BSES-SF^m^ (14-70), MBFES^n^ (30-150) at 1 week. Secondary: exclusive breastfeeding (EBF) status and feeding categories per the World Health Organization.	Group assignment (MR+ standard vs standard) → maternal self-efficacy, maternal satisfaction, and EBF at 1 week.

a VR: virtual reality.

b VAS: Visual Analog Scale.

c PHQ-9: Patient Health Questionnaire-9.

d GAD-7: Generalized Anxiety Disorder-7 Scale.

e PSS: Perceived Stress Scale.

f WHOQOL-BREF: World Health Organization Quality of Life–BREF.

g PRAQ-20: Pregnancy-Related Anxiety Questionnaire.

h FOC: fear of childbirth.

i PSUS: Pictorial System Usability Scale.

j SSQ: Speech, Spatial and Qualities of Hearing Scale.

k IVE: immersive virtual environment.

l STAI: State-Trait Anxiety Inventory.

m BSES-SF: Breastfeeding Self-Efficacy Scale-Short Form.

n MBFES: Maternal Breastfeeding Evaluation Scale.

**Table 5.** Results, conclusion, and implications for future research*.*

Study	Results	Conclusions	Implications for future research
Xie and Zeng [23]	No significant difference in second-stage labor duration between groups. The treatment group had significantly lower postpartum hemorrhage (151.28 mL vs 248.95 mL; *P*=.008). Pain scores were significantly lower in the treatment group (3.73±1.87 vs 5.97±2.66; *P*<.05). No significant effect on neonatal Apgar scores or weight. The treatment group had a higher rate of intact perineum and lower rates of severe perineal lacerations (*P*<.05). Anxiety scores were significantly lower in the treatment group during labor (*P*<.001). Self-efficacy and quality of life satisfaction were significantly higher in the treatment group (*P*<.001).	VR^a^ technology combined with moderate perineal protection can improve childbirth outcomes by reducing pain, anxiety, and perineal trauma. This approach increases maternal confidence and promotes a better childbirth experience without adverse effects on newborns. The intervention can be widely applied in clinical obstetric settings.	Further studies should use standardized psychological scales to assess maternal mental health. Future research should evaluate long-term maternal and neonatal outcomes. Expanding VR health education to other aspects of maternal care, such as postpartum recovery, may be beneficial.
Lee et al [34]	Significant reduction of depressive and anxiety symptoms comparing before and after VR program. Quality of life scores significantly increased. No significant change in perceived stress scores. The program was particularly effective in the high-risk subgroup with severe depression and anxiety.	The VR intervention was most effective for Cluster 2, which had the most severe mental health issues. Depression, anxiety, and quality of life improved significantly in the high-risk depression group (PHQ-9^b^≥8). Participants with high anxiety (GAD-7^c^≥5) showed improvements, but they were not statistically significant. Perceived stress did not improve significantly in any of the high-risk groups.	Future studies should customize VR interventions for specific high-risk subgroups. A longer intervention period (more than 5 weeks) may be needed to show stronger effects. VR programs should incorporate stress management techniques to improve perceived stress outcomes.
Caballero-Galilea et al [40]	Significant postintervention decreases in PRAQ-20^d^ anxiety scores (*P*<.001); greater effect among younger and primiparous women; no adverse effects; participants reported increased confidence and preparedness.	Immersive VR childbirth simulation effectively reduced pregnancy-related anxiety, was well-tolerated, and perceived as supportive; results support VR as a complement to traditional prenatal education.	Recommend RCTs^e^ and longitudinal studies to confirm effects, assess long-term outcomes, explore emotional mechanisms, and adapt VR childbirth education for diverse populations.
Siivola et al [41]	Users successfully navigated the VR program with minimal instructions. No motion sickness was reported. Users preferred using the program on personal devices rather than in research settings. The program was perceived as effective for childbirth education. Some improvements were suggested, such as adjusting camera angles and adding more immersive birth sequences.	The VR CBE^f^ program was well received and demonstrated potential for improving childbirth education. It provided a realistic and immersive experience, making learning more engaging. Further studies are needed to assess its impact on reducing FOC^g^.	Future studies should test the VR CBE with pregnant women and individuals diagnosed with FOC. Evaluating the effectiveness of VR-based learning in improving childbirth preparedness. Exploring further enhancements, such as interactive VR environments.
Siivola et al [42]	Users watched an average of 33 minutes of VR content. 4/5 participants watched all 18 videos; one skipped a few due to prior knowledge. Usability scores were high (87 for VR and 76 for independent testing). VR improved learning outcomes compared to traditional CBE. Fear of childbirth impact varied; one participant’s FOC increased, while another’s FOC decreased.	VR CBE provided a realistic, immersive learning experience. The program was well-received, particularly for first-time parents.	Testing with a larger and more diverse sample. Expanding VR content to include more childbirth scenarios (eg, medical pain relief and home births). Investigating long-term retention of knowledge gained through VR CBE.
Tang et al [43]	Participants found the simulation engaging and realistic. The simulated baby’s behavior (delayed latching and hunger cues) effectively conveyed real breastfeeding challenges. Some participants felt frustrated by the lack of clear guidance and suggested integrating more explicit game elements, such as tutorial instructions and achievement notifications. The simulation was seen as a valuable tool for antenatal education, facilitating discussions between parents and health care providers.	VR can effectively simulate breastfeeding challenges, promoting awareness and reflection. Participants expected game-like elements within the VR environment, raising questions about balancing realism and usability in health care simulations. The lack of explicit feedback within the simulation led to user frustration, suggesting the need for guided interactions.	Investigate the optimal balance between realism and playfulness in VR health care simulations. Explore the impact of gamification on learning outcomes in antenatal education. Assess the long-term effectiveness of VR breastfeeding simulations in improving breastfeeding success rates.
Tang et al [44]	Thematic analysis revealed 2 dominant themes. The first theme highlighted the emotional and logistical imbalance often present in breastfeeding relationships, where one parent typically assumes the feeding role while the partner provides indirect support. Participants frequently emphasized the importance of practical and emotional presence from partners, such as preparing food, staying close during night feeds, or simply offering words of encouragement. The second theme focused on parents’ evolving understanding of breastfeeding. Many participants described the initial stages as marked by uncertainty, technical difficulties, and emotional strain. Public breastfeeding emerged as a significant source of stress, with participants reporting a lack of appropriate facilities and discomfort associated with social exposure. Nevertheless, some individuals reported that prior exposure to breastfeeding scenes, such as observing siblings or relatives breastfeed, had helped normalize the practice and reduced their own discomfort. Participants expressed preferences for visually clean, aesthetically pleasing environments rendered in high-fidelity 3D graphics, along with gender-neutral color palettes and customizable avatars. Furthermore, they emphasized the value of including realistic but non-overwhelming challenges within the simulation. Based on these findings, the researchers refined the VR simulation in the third phase. The updated version included 3 scenarios: breastfeeding in a home setting with a partner present, a public park environment populated with distant nonplayer characters and background noise, and a workplace scenario simulating a return to work, with social pressure and unexpected interruptions. The virtual baby was also redesigned to incorporate feeding behaviors, latching mechanics, and emotional states, represented through facial expressions and audio cues. A 9-state behavior model was implemented to simulate the unpredictability of infant behavior during feeding. Thematic analysis of the interview transcripts revealed 2 overarching themes. The first described participants’ deep affective engagement with the simulation. Many users, especially those with prior breastfeeding experience, reported that the simulation closely mirrored their own lived realities, triggering emotional recall, moments of joy and frustration, and a sense of bonding with the virtual baby. The second theme referred to aspects of the simulation that disrupted immersion. These frictions stemmed from mismatches between the users’ bodies and their virtual avatars, insufficient interactivity with nonplayer characters, overly pristine environments that did not reflect the reality of a household with a newborn, and the absence of tactile or haptic feedback. Additionally, several participants indicated a desire for more explicit feedback mechanisms, such as prompts or tutorials, to guide them through the feeding process. The simulation was generally perceived as nonthreatening and emotionally safe. None of the participants reported feeling discouraged or overwhelmed; rather, the VR experience facilitated nuanced reflection on breastfeeding and helped both parents and partners consider their roles more deeply.	VR breastfeeding simulations can enhance antenatal education by providing immersive, realistic experiences. The study highlighted challenges in VR design, such as managing uncertainty, realism, and user expectations. In summary, the study’s results indicate that interactive VR simulations can effectively communicate the complexities of breastfeeding by combining emotional engagement with experiential learning. Rather than providing explicit instruction, such systems may be most impactful when they offer opportunities for introspection and encourage users to reframe their expectations. The findings underscore the importance of designing with empathy, realism, and flexibility in mind—particularly when addressing sensitive and deeply personal aspects of early parenthood.	Future work should explore: Customization options to reflect diverse family structures and breastfeeding experiences. Incorporation of haptic feedback to simulate the physical aspects of breastfeeding. Longitudinal studies on the impact of VR simulations on breastfeeding success.
Noben et al [45]	There was an increase in the Visual Analog Scale for Anxiety (VAS-A) score between the first and second measurements of 1.5 cm for the women in the VR group compared to 0.8 cm for women in the control group (95% CI −0.1 to 2.0; *P*=.08). For their partners, there was an increase of 1.4 cm in the VR group compared to 0.9 cm in the control group (95% CI −0.5 to 1.6; *P*=.30). The following variables showed a significant relation with ΔVAS-A: baseline VAS-A (*F*_1,75_=8.4; *P*=.01) and history of CD^h^ (*F*_1,75_=6.0; *P*=.02). The increase in the baseline VAS-A score at time point 2 (at admission) in women in the VR group with a history of emergency CD was 1.7 cm smaller than that in women with a history of emergency CD in the control group, although this effect was not significant (*P*=.06). Median scores on the SSQ^i^ for motion sickness symptoms reflected the absence of discomfort caused by the VR video. There was no significant difference in scores on the Tilburg Pregnancy Distress Scale subscales for both time points 1 and 2 between the VR group and the control group. Individuals in the VR group without a history of emergency CD perceived a higher quality of care than the control group (10.2, SD 3.8 vs 12.9, SD 3.5; *P*=.02). 15% of women responded that they did not feel more prepared after seeing the VR video. 85% of women responded positively. From the partners, 79% responded positively. The 21% partners did not feel more prepared after seeing the VR video.	The study did not show a decrease in preoperative anxiety after VR information provision for patients undergoing elective CD. There was a trend toward decreased preoperative anxiety in the subgroup of women with a history of emergency CD who watched the VR video.	Further research for identifying the characteristics of subgroups of patients who would potentially benefit from VR information provision is necessary.
Setiawan et al [46]	The results of checking the vital signs after doing exercise with VR showed no sign of bad health conditions. The average of all aspects of the IVE^j^ questionnaire given is 4.26. (max 5)	The average results of all aspects of the components in the IVE questionnaire showed positive results. That indicates the VR application for pregnancy exercise is feasible to use by pregnant women.	In future research, it is possible to develop mixed reality applications for pregnancy exercise so that when doing exercise, pregnant women can still see the environment in the real world.
Park and Kim [47]	State Anxiety: Experimental group: mean 44.53 (SD 7.58); Control group: mean 52.93 (SD 8.96); *P*=.009. VR intervention significantly reduced anxiety levels. Stress Related to Preterm Labor: Experimental group: mean 48.66 (SD 4.82); Control group: mean = 55.87 (SD 6.91); *P*=.002. VR education significantly lowered stress scores. Uterine contractions: frequency (times/min): Experimental: 1.00 (SD 0.75); Control: 1.84 (SD 0.76); *P*=.004. Intensity (mm Hg): Experimental: 10.13 (SD 3.97); Control: 23.43 (SD 10.11); *P*<.001. Significant reductions in both frequency and strength of uterine contractions in the experimental group. Cervical length: Experimental group: 33.93 (SD 5.33) mm; Control group: 31.62 (SD 7.38) mm; *P*=.009. Cervical length increased more in the experimental group, indicating a possible delay in labor progression. Pregnancy health care practice behavior: Experimental group: 71.60 (SD 5.86); Control group: 67.37 (SD 5.47); *P*=.047; improved health-promoting behaviors in the experimental group. Self-efficacy in pregnancy health care: Experimental group: 46.93 (SD 2.93); Control group: 40.81 (SD 4.98); *P*=.001. Higher confidence in managing pregnancy in the VR group.	The VR-based prenatal education program was statistically and clinically effective in improving: Psychological well-being (reduced anxiety and stress). Physiological indicators (lower uterine activity and increased cervical length). Behavioral outcomes (better self-care practices and self-efficacy).	Future studies should: Replicate the intervention across multiple institutions, diverse geographic areas, and larger sample sizes to confirm its effectiveness and improve external validity. Use RCTs to strengthen causal inferences and minimize confounding factors. Explore personalized VR content tailored to clinical severity (eg, cervical length, prior preterm labor) and educational needs. Involve interdisciplinary collaborations (eg, obstetrics, psychology, digital health, and user experience design) to enhance both content relevance and technological usability. Assess the sustained impact of VR-based education on: preterm birth rates, neonatal outcomes, maternal mental health postpartum, and long-term health behavior adherence. Examine: barriers to use (eg, motion sickness and device usability); user interface improvements; low-cost and mobile-accessible versions for wider clinical implementation. Include mediator and moderator analysis to isolate which components of the intervention are most impactful.
Montoya-Moncada et al [48]	No between-group differences (BSES-SF^k^ mean 63.31 vs 63.10; *P*=.87; MBFES^l^ 133.48 vs 134.03; *P*=.84). Overall EBF^m^ at 1 week = 93.1%; distribution by arm 50%/50%.	MR adjunct not superior within 1-week window; very high early EBF suggests strong baseline supports.	Larger samples, multiple sessions, longer follow-up; assess usability and accessibility and consider integration with postnatal supports.

a VR: virtual reality.

b PHQ-9: Patient Health Questionnaire-9.

c GAD-7: Generalized Anxiety Disorder Scale-7.

d PRAQ-20: Pregnancy-Related Anxiety Questionnaire.

e RCT: randomized controlled trial.

f CBE: childbirth education.

g FOC: fear of childbirth.

h CD: cesarean delivery.

i SSQ: Speech, Spatial and Qualities of Hearing Scale.

j IVE: immersive virtual environment.

k BSES‑SF: Breastfeeding Self‑Efficacy Scale – Short Form.

l MBFES: Modified Breastfeeding Self-Efficacy Scale.

m EBF: exclusive breastfeeding.

**Table 6.** Hardware and software equipment and virtual reality content.

Study	Hardware and software equipment	Virtual reality contents
Xie and Zeng [23]	Hardware: desktop VR^a^ system. Software: SpaceMax VR software for interactive 3D labor room simulation.	3D interactive virtual labor room environment. Virtual scenarios of delivery processes and perineal protection techniques. Realistic depictions of hospital settings, medical staff interactions, and childbirth procedures.
Lee et al [34]	No specific details provided, but VR technology was used to create an immersive training and mental health promotion program.	The VR program included 4 modules: Module 1: Game-Based Cognitive Training. Module 2: Advanced Game-Based Challenge. Module 3: Psychoeducation and Emotional Support. Module 4: Memory and Anticipation Training.
Caballero-Galilea et al [40]	Hardware: Meta Quest 3 HMD^b^; obstetric props for kinesthetic immersion. Software: custom Unity 3D simulation of eutocic childbirth with interactive hospital environment and soundscapes.	Immersive VR simulation of eutocic childbirth in a virtual hospital setting, guiding participants through admission, monitoring, pain management, epidural, active labor, delivery, and immediate postpartum with multisensory cues and gaze-based interaction; aimed at realistically reproducing the childbirth experience, without a structured psychological or anxiety-focused educational component.
Siivola et al [41]	Hardware: Insta360 Pro camera, Oculus Quest 2 VR headset, and Zoom H6 recorder. Software: Adobe Premiere Pro, Pano2VR, and HandBrake.	360° multimedia childbirth program including panoramic and video-based labor scenarios, supportive audio clips, brief text and image content, and web resources covering the birth environment, pain relief options, labor positions, monitoring, comfort strategies, and birth preferences.
Siivola et al [42]	Hardware: Oculus Quest 2 VR headset and Insta360Pro camera. Software: Editing: Adobe Premiere Pro and HandBrake (for video compression) VR Development: Pano2VR Accessibility: hosted on a website accessible via multiple devices (VR headsets, tablets, smartphones, and computers).	360° videos depicting hospital environments and birth scenarios. Audio narration explaining childbirth procedures. Interactive elements to explore different birthing techniques. Additional multimedia content (text, images, and sound clips).
Tang et al [43]	Hardware: Oculus Rift CV1, Leap Motion Controller, and tangible probe representing a baby (tracked using Oculus Touch controllers). Software: unity-based VR simulation, SDKs from Oculus and Leap Motion, and FABRIK inverse kinematics.	VR breastfeeding simulation across home, public, and workplace settings, using a self-avatar and responsive virtual baby to practice latching and feeding under realistic social and environmental conditions; interaction was minimally guided and deliberately nongamified to preserve realism.
Tang et al [44]	Oculus Rift CV1 VR headset. Oculus Touch controllers. Leap Motion Controller. Tangible baby representation (plush dolls weighted to simulate a newborn).	The simulation included 3 scenarios: Personal living space: a home setting for private breastfeeding. Public park: outdoor breastfeeding experience with environmental influences. Meeting room: breastfeeding in a workplace setting.
Noben et al [45]	Informed app and VR glasses	The 360° VR video shows all the aspects of a CD, including the admission on the ward, the operating room, placement of spinal analgesia, and the birth of the baby when the gynecologist lifts the baby above the sterile environment.
Setiawan et al [46]	Xiaomi Redmi Note 4 smartphone and VR Shinecon.	VR prenatal exercise program with a preprogrammed virtual trainer delivering 10 sessions (warm-up, core seated pregnancy exercises, and cool-down) focused on safe stretching, pelvic movements, and breathing for childbirth preparation, with simple head-nod interaction in an immersive natural setting.
Park and Kim [47]	VR head-mounted display: equipped with finger motion recognition for navigating menus and selecting content without the need for physical controllers. 360-degree camera.	VR intervention combining educational modules on preterm labor, prenatal care, symptom monitoring, hospitalization, and discharge with relaxation and meditation sessions using nature-based imagery, breathing guidance, and nurse-led narration to support knowledge, stress reduction, and emotional regulation.
Montoya-Moncada et al [48]	MR headset; silicone breast model; infant doll; neonatal simulator; immersive video playback via VR Video Player.	Immersive breastfeeding scenario videos shown via headset as part of the guided session.

a VR: virtual reality.

b HMD: head-mounted display.

### Data Analysis

We conducted a thematic analysis following Braun and Clarke 6-phase framework [49] to synthesize qualitative and mixed methods insights across the included studies. This approach encompassed the phases of familiarization with the data, generating initial codes, searching for and reviewing themes, defining and naming themes, and producing the final report. Our main objective was to identify patterns related to participant experiences, perceived impacts, and implementation characteristics of XR-based interventions. More specifically, during the familiarization phase, 2 researchers (SP and ONM) thoroughly read the methodology, results, discussion, and conclusion sections of each included study. In the initial coding phase, we systematically labeled and coded relevant segments of the data, identifying features pertinent to our research focus. During the theme generation phase, the coded data were organized into candidate themes. In the reviewing themes phase, we refined and consolidated the themes through an iterative process of comparison, discussion, and evaluation of the coherence and distinctiveness of each theme against the preliminary coded data. Subsequently, in the defining and naming themes phase, we finalized the themes by articulating the scope and content of each one. Finally, in the reporting phase, we described the thematic findings and their relative prevalence across the dataset.

Throughout the analysis, we used an inductive, data-driven approach, allowing the themes to emerge organically from the data without reliance on preexisting theoretical models [50]. In parallel, we complemented this bottom-up strategy with a top-down perspective: the extraction and interpretation of findings were also guided by the research questions formulated during the scoping review process.

## Results

### Data Charting Process and Data Items

We included 11 studies [23,34,40-48] (Figure 1). A data extraction template has been deployed to outline the relevant data corresponding to the research objectives. The data, recorded either in narrative form or as nominal values, included the variables indicated in Tables 2-6.

To provide a clearer overview of the findings, the “Results” are structured according to the 5 research questions defined in the “Introduction.” Each subsection presents a synthesis of evidence addressing the corresponding question.

### RQ1: What Are the Primary Applications of XR in Prenatal Education Programs?

To address RQ1, the included studies were analyzed to identify how virtual, augmented, mixed, and immersive technologies were applied within prenatal, childbirth, and early parenting education. The selected 11 studies included in the final review [23,40-48], from 2019 to 2025, and conducted across 8 countries (China [23], Korea [34,47], Spain [40], Finland [41,42], Belgium [43-45], Netherlands [46], Indonesia [47], and Colombia [48]). The works examined the use of XR technologies, mainly VR and 360° video-based simulations, for prenatal, childbirth, and breastfeeding education, as well as for promoting mental health, relaxation, and physical activity during pregnancy (Table 2). Most studies were single-center and focused on pregnant women as the target population, with 3 studies also involving partners [43-45]. Study designs included RCTs [23,45,48], quasi-experimental or interventional studies [34,40,46,47], pilot or feasibility studies [41,42,46], and qualitative user-centered design investigations [43,44]. Sample sizes ranged from small qualitative cohorts (from 10 to 25 participants) to larger controlled studies (from 60 to 200 participants). From a technological point of view, interventions used a range of immersive solutions: (1) fully immersive VR HMDs such as Oculus Quest, Rift, or equivalent [34,40-47]; (2) 360° or 3D video-based environments accessible through computers or mobile devices [23,41,42]; (3) hybrid setups integrating interactive elements, soundscapes, and guided relaxation content [40,43,47]; and (4) a MR headset used with a silicone breast model and infant doll to simulate breastfeeding [48]. Several studies applied structured instructional or design frameworks such as the Analyze, Design, Develop, Implement, and Evaluate model (ADDIE model) [47] and the IMCHB [47], or used user-centered and co-design methodologies [41-44].

### RQ2: What Benefits Have Been Reported Regarding the Use of XR in Prenatal Education?

#### Overview

The included studies evaluated a range of psychological, physiological, educational, and experiential outcomes: (1) anxiety reduction and mental health promotion [34,40]; (2) childbirth education and fear-of-childbirth management [41,42]; (3) prenatal care for hospitalized or high-risk pregnancies [50]; (4) breastfeeding skill development and experiential learning [43,44,48]; (5) prenatal exercise motivation and maternal physical well-being [46]; (6) pain management and obstetric outcomes during labor [23]; and (7) reduction of preoperative anxiety before cesarean delivery [45]. More in detail, the reported outcomes were categorized into four main domains: (1) psychological outcomes (depression, anxiety, FOC, and perceived stress); (2) physiological outcomes (pain reduction, perineal trauma, postpartum hemorrhage, uterine contractions, and cervical length); (3) educational outcomes (knowledge acquisition, memory retention, and perceived preparedness); and (4) experiential outcomes (user experience, usability, acceptability, and immersion).

#### Psychological Outcomes

Anxiety reduction was consistently reported across multiple studies. Lee et al [34] conducted a 5-week VR-assisted mental health program for pregnant women, reporting significant reductions in depressive and anxiety symptoms (Patient Health Questionnaire-9 [PHQ-9] and Generalized Anxiety Disorder-7 [GAD-7]) and improvements in quality of life (WHOQOL-BREF [World Health Organization Quality of Life–BREF]). No statistically significant changes were observed in Perceived Stress Scale (PSS) scores, suggesting that while the VR program effectively enhanced emotional well-being, it may not directly influence stress-related factors. The intervention appeared particularly beneficial for participants at higher baseline risk for depression and anxiety. Similarly, Caballero-Galilea et al [40] found that controlled VR exposure to the childbirth process reduced pregnancy-related anxiety in third-trimester women. Noben et al [45] evaluated the use of 360° VR videos to prepare women and their partners for elective cesarean delivery. Although the intervention did not significantly reduce overall preoperative anxiety compared with standard information provision, most participants, both women and partners, reported feeling more prepared and less fearful about the upcoming procedure. Qualitative feedback indicated that the immersive visualization of the operating room and surgical process fostered familiarity and reduced uncertainty, particularly among those with prior emergency cesarean experience. Thus, while VR did not achieve a statistically significant decrease in anxiety scores, it enhanced perceived preparedness and satisfaction, suggesting potential benefits in specific subgroups. Xie et al [23] showed that women receiving VR-based prenatal education combined with perineal protection reported significantly lower anxiety. FOC was a specific target in 2 studies [41,42], both of which reported reductions in FOC levels and positive user feedback on emotional preparedness after participation in VR-based childbirth education. Park and Kim [47] conducted an experimental study targeting pregnant women hospitalized for preterm labor. The intervention group who received VR-based prenatal education demonstrated statistically significant improvements in self-efficacy, pregnancy-related knowledge, and emotional stability, along with a higher perceived usefulness of the training content compared to the control group. The VR program provided tailored content that addressed psychological well-being during hospitalization and included simulations of childbirth and newborn care. Importantly, participants in the VR group showed lower levels of anxiety and stress and greater readiness for labor and postpartum care.

To summarize, 9 out of 11 (81.8%) studies [23,34,40-48] assessed psychological or emotional dimensions such as anxiety, depression, perceived stress, or FOC. Among these, 7 [23,24,40-42,45,47] out of 11 studies [23,34,40-48] (63.6%) reported statistically significant or qualitatively positive effects on anxiety or related constructs. Specifically, studies [23,34,40,45] reported measurable anxiety reduction following exposure to immersive prenatal or perinatal experiences. Siivola et al [41,42] observed reductions in FOC and enhanced emotional readiness, while Park et al [47] found decreased stress and improved emotional stability in hospitalized women receiving VR-based prenatal education. One study [46] focused primarily on physical activity and did not measure mental health outcomes, whereas another [44] reported mixed results, some participants described VR breastfeeding simulations as “emotionally demanding” or “challenging,” suggesting that immersive realism can occasionally elicit discomfort alongside learning benefits. Overall, these findings suggest that immersive XR interventions could have an impact on enhancing psychological well-being, though emotional responses are related to the realism and sensitivity of the simulated content.

#### Physiological Outcomes

Some studies incorporated physiological or behavioral measures to complement psychological outcomes. VR-assisted prenatal preparation was associated with lower maternal heart rate, reduced pain scores, and higher rates of perineal integrity during childbirth [23]. Setiawan et al [46] monitored vital parameters before and after each VR-based exercise session, observing no adverse cardiovascular responses and confirming the feasibility of moderate physical activity guided through immersive environments. Similarly, Park et al [47] reported that VR relaxation modules stabilized uterine activity among women hospitalized with threatened preterm labor, contributing to improved comfort and adherence to treatment. Across all studies, no serious adverse effects were observed; minor discomfort, such as transient dizziness or eye fatigue, was infrequently reported and self-resolving. To summarize, 3 (30%) studies [23,46,47] reported physiological or obstetric indicators, either as primary or secondary outcomes. Among them, Xie et al [23] documented significant improvements in childbirth-related parameters, including reduced pain scores, higher rates of intact perineum, and shorter labor duration in the VR-assisted group. Park et al [47] observed stabilization of physiological stress markers such as systolic blood pressure, heart rate, and uterine activity. Setiawan et al [46] confirmed the safety and feasibility of VR-guided exercise, with no adverse cardiovascular responses. No study reported serious side effects, and among the few studies that assessed VR-related tolerability, adverse effects were generally absent or limited to minor, transient complaints. Noben et al [45] reported no discomfort or motion sickness, Setiawan et al [46] found no postsession nausea, vomiting, or dizziness, and Siivola et al [41] reported no motion sickness during testing. Minor transient adverse events were documented in only 2 studies (<20%): Siivola et al [42], in which 1 participant reported eye strain and another minor HMD-related discomfort, and Park et al [47], which reported 1 withdrawal due to HMD-related discomfort. Overall, most studies did not systematically assess VR-related side effects.

#### Educational Outcomes

Educational impact was evaluated through measures of knowledge acquisition, engagement, motivation, and self-efficacy. Siivola et al [41,42] developed and tested immersive childbirth education programs using 360° videos and VR headsets. Participants consistently rated the experiences as usable, realistic, and engaging, with reported improvements in childbirth knowledge and preparedness. However, evidence regarding the FOC reduction remains inconclusive. Tang et al [43,44] found that VR breastfeeding simulations supported experiential learning through embodied interactions, enabling users to visualize and practice realistic feeding challenges in a safe context. However, a subset of users reported frustration with the lack of explicit feedback or structured guidance during the simulation. Montoya-Moncada et al [48] found no statistically significant differences between the MR-based educational strategy and traditional breastfeeding counseling in terms of maternal self-efficacy and satisfaction. Park et al [47] highlighted that immersive prenatal modules improved engagement, comprehension, and emotional connection to content, aligning with the principles of the IMCHB model. Setiawan et al [46] demonstrated that VR exercise programs for pregnant women are feasible and could positively impact adherence to physical activity, suggesting behavioral reinforcement potential through gamified, self-paced environments. Overall, 6 [41-44,46,47] out of 11 studies [23,34,40-48] (54.5%) explicitly evaluated educational end points, such as knowledge acquisition, preparedness for childbirth, self-efficacy, or skill transfer. The studies [41-44,47] reported improvements in knowledge retention, confidence, and comprehension, particularly when interactive or 360° scenarios were used. Setiawan et al [46] found increased motivation and adherence to prenatal exercise programs, suggesting educational reinforcement through gamified feedback. Conversely, Caballero-Galilea et al [40] observed reductions in anxiety but did not detect significant changes in perceived knowledge, and one breastfeeding simulation study [44] noted that while participants valued the realism, they sought clearer instructional guidance. Approximately two-thirds of the included studies demonstrated enhanced learning or self-efficacy outcomes, though some highlighted the need for improved instructional design within immersive systems.

#### Experiential Outcomes

User-centered evaluations were prominent in at least 6 (60%) studies [41,42,44-47], which mainly investigated usability, comfort, and motion sickness. Most users reported high satisfaction and found the VR interfaces intuitive, with minor reports of motion discomfort. Studies reported user-centered or experiential metrics, including usability, acceptability, immersion, and motion sickness. Across these, most participants described immersive experiences as engaging, realistic, and emotionally meaningful. Some studies [41-44] emphasized the positive influence of interactivity and presence on emotional engagement. Other [44] noted occasional discomfort or cognitive overload among participants unfamiliar with VR interfaces. Reports of motion sickness were infrequent and mild.

Overall, user experience outcomes were favorable in the included studies, supporting the feasibility and acceptability of immersive prenatal and perinatal education.

When aggregated, 9 [23,34,40-42,45-48] of 11 studies [23,34,40-48] (81.8%) reported at least 1 positive outcome domain, and 6 [23,34,41,43,46,48] of 11 [23,34,40-48] (54.5%) demonstrated statistically significant improvements in at least one primary variable (eg, anxiety, pain, knowledge, or satisfaction). Psychological and educational benefits were the most consistent, particularly in anxiety reduction and learning engagement, although results were heterogeneous for FOC and preoperative anxiety. Physiological and experiential outcomes showed promising but preliminary evidence of safety and acceptability. Taken together, these findings reinforce the potential of XR technologies, especially VR, to enhance emotional readiness, experiential learning, and patient engagement during pregnancy and childbirth, while highlighting the need for larger, standardized, and multicenter studies to validate these early results. Tables 2-6 present a comprehensive overview of the included studies, summarizing study characteristics, participant demographics, methodological features, key results and implications, and details of the educational programs, including technological components and content design.

### RQ3: What Challenges and Limitations Are Identified in the Integration of XR Into Prenatal Courses?

RQ3 examined the methodological diversity and implementation challenges reported in the included studies. As summarized in Table 3, methodological heterogeneity was observed across the included studies, reflecting the exploratory nature of research on immersive and XR technologies in prenatal and childbirth education. While some studies [23,34,40,45,47] adopted controlled or quasi-experimental frameworks to assess measurable outcomes, such as anxiety reduction or physiological responses, others relied on exploratory or design-based methodologies to examine user perceptions, usability, or content development processes. This diversity illustrates the coexistence of intervention trials and formative research, each contributing different types of evidence to the field. Differences also emerged in the exposure design and delivery parameters. The duration and frequency of immersive experiences varied considerably, from single brief sessions focusing on emotional regulation or preoperative preparation to multiweek interventions structured as comprehensive educational programs. The level of immersion also ranged widely, encompassing fully immersive VR headsets, semi-immersive 360° video environments, and hybrid configurations integrating soundscapes, narration, or limited interactivity. The measurement tools and outcome frameworks were equally inconsistent. Psychological outcomes such as anxiety, depression, or FOC were assessed with different validated instruments [eg, the State-Trait Anxiety Inventory (STAI), the Wijma Delivery Expectancy/Experience Questionnaire (W-DEQ), and the Edinburgh Postnatal Depression Scale (EPDS)], which hinders cross-study comparison. In contrast, educational and experiential outcomes were often measured through ad hoc questionnaires or qualitative interviews, limiting external validity but providing valuable insight into user engagement and emotional resonance. Some quantitative studies applied inferential statistics to compare intervention and control groups, whereas qualitative research emphasized thematic synthesis or user-centered evaluation frameworks. Follow-up assessments were infrequently conducted, reducing the capacity to assess long-term retention or behavioral impact. Most studies recruited only women, with sample sizes ranging from 10 participants in qualitative co-design studies [44] to 200 participants in randomized clinical trials [23]. Eligibility criteria were often highly restrictive, for example, targeting primiparous women without complications [23] or patients scheduled for elective cesarean delivery [45]. Such selectivity, while enhancing internal validity, reduces generalizability and further contributes to the methodological heterogeneity observed across the field. Intervention structures also differed markedly. For instance, Lee et al [34] implemented a progressive multimodule VR mental health program, Setiawan et al [46] integrated pre- and postsession physiological monitoring during VR exercise, and Park et al [47] developed a guided prenatal education model using the IMCHB framework for hospitalized women. This diversity of methodological orientations, ranging from structured trials to exploratory pilot studies, reflects both the experimental stage and the rapid evolution of immersive applications in prenatal education. Despite increasing recognition of coparenting and partner involvement, only 3 studies [43-45] explicitly included partners. This underscores an underexplored area for future research on shared learning and emotional coregulation during the perinatal period. Equity and accessibility were addressed in a limited number of studies. Barriers such as digital literacy, comfort, device availability, and motion sensitivity were acknowledged as potential obstacles to the equitable adoption of immersive education. Notably, the inclusion of multidevice access options (eg, VR headset, tablet, or smartphone [41,42,47]) was viewed positively by users, emphasizing the importance of flexible and inclusive design approaches for diverse populations. Overall, the methodological heterogeneity across studies underscores both the innovation potential and the fragmentation of current immersive technologies research in prenatal care. The field would benefit from shared methodological guidelines, standardized reporting criteria, and mixed methods designs that integrate psychological, educational, and experiential dimensions within unified evaluation frameworks.

### RQ4: What Methodologies and Technological Approaches Are Commonly Used in Studies on XR for Prenatal Education?

RQ4 analyzed the technological and design characteristics of XR interventions, including hardware, software, and the structure of the immersive content. Technological diversity was substantial across studies, reflecting the evolving and experimental nature of immersive technological applications in maternal health. Most interventions used HMDs such as Oculus Rift, Oculus Quest, or smartphone-compatible VR viewers [34,41-44,46,47], allowing full or semi-immersive 3D visualization of childbirth or breastfeeding scenarios. Others implemented 360° panoramic videos accessible via computers, tablets, or mobile devices to broaden accessibility and minimize cybersickness [41,42,45].

Software environments were typically developed using Unity (Unity Technologies) or Pano2VR (Garden Gnome Software), integrated with supplementary editing tools such as Adobe Premiere Pro (Adobe Inc) and HandBrake (HandBrake Team) for video compression and cross-platform usability [41-43].

Across studies, immersive content was structured around thematic educational modules:

- Childbirth process simulations, including stages of labor, pain management, and hospital familiarization [23,41,42].
- Mental health and relaxation modules, focusing on stress reduction, guided breathing, and emotional resilience [34,47].
- Skill-based training, such as breastfeeding interaction scenarios and prenatal physical exercise routines [43,44,46,48].

Most interventions combined visual, auditory, and interactive cues to enhance engagement and learning transfer. Some included embodied tasks, for example, hand motion tracking, tactile interaction with a doll and/or silicone breast model [43,44,48], or guided voice-over narration simulating clinician or midwife presence [47]. User experience design was generally informed by user-centered frameworks and pedagogical models such as the IMCHB model and constructivist learning theory, emphasizing experiential learning through realistic simulation [47].

Overall, immersive technologies demonstrated versatility in integrating multisensory and interactive components adaptable to both clinical and home environments. However, variation in technical specifications and delivery platforms underscores the current lack of standardization in immersive maternal education tools.

### RQ5: What Research Gaps Exist in This Area, and What Future Directions Are Recommended?

First, modest sample sizes and narrow eligibility criteria (eg, low-risk, primiparous women or inpatient cohorts) limit generalizability and constrain inferences about the applicability of XR. Only 3 studies enrolled partners [43-45], despite growing evidence in favor of shared parental education. Second, heterogeneity in study designs and measurement approaches hampers cross-study comparisons and meta-analytic synthesis. Psychological outcomes were often captured with validated scales, whereas experiential and usability outcomes frequently relied on ad hoc instruments. Standardized, validated tools tailored to XR-based maternal education remain lacking. Technologically, few interventions collected objective usability metrics (eg, engagement time and motion-tracking data), and accessibility testing for participants with low digital literacy or limited device access was rarely undertaken [41,42]. Moreover, ethical and equity considerations, including data privacy, the digital divide, and cultural adaptation of immersive content, were seldom discussed, despite their importance for global health implementation. Additional work should examine the suitability of mixed-reality tools across diverse populations, particularly in low- and middle-income countries where access to innovative educational strategies may be constrained yet potentially highly beneficial [48]. Future research should deploy RCTs that use more intensive educational strategies, incorporate multiple sessions, and extend follow-up to clarify long-term effects on maternal self-efficacy, satisfaction, and exclusive breastfeeding rates [48]. Finally, there remains a pressing need for longitudinal and comparative studies that evaluate behavioral and clinical outcomes beyond the immediate postpartum period. Researchers should explore scalable, low-cost immersive solutions and hybrid learning models that combine simulation with interpersonal support. Collaborative, interdisciplinary efforts, linking clinicians, designers, and educators, are essential to establish best practices and ensure safe, inclusive integration of XR technologies into prenatal and childbirth education.

### Overall Summary of Findings

Overall, the synthesis across RQ1-RQ5 indicates that immersive and XR technologies are being increasingly integrated into prenatal and childbirth education with encouraging outcomes across psychological, physiological, educational, and experiential domains. Despite methodological heterogeneity and small sample sizes, most studies demonstrated positive effects on anxiety reduction, learning engagement, and emotional preparedness. However, limited standardization in design, measurement, and accessibility testing highlights the early developmental stage of this field. These results collectively support the feasibility and potential of XR as an innovative complement to conventional prenatal education, while underscoring the need for rigorous, inclusive, and longitudinal research to consolidate its evidence base.

### Thematic Analysis

A total of 5 major themes and the related subthemes emerged from the analysis. To further contextualize these patterns, we have integrated responses to the 5 guiding research questions underpinning this review, supported by evidence from the included academic literature. Proportions refer to the 11 included studies [23,34,40-48].

#### Theme 1: VR as a Catalyst for Emotional, Cognitive, Physical, and Physiological Outcomes

This theme explores how immersive experiences promote emotional readiness, self-efficacy, and physiological comfort during pregnancy and childbirth. Across 8 [23,34,40,42,45-48] of 11 [23,34,40-48] (72.7%) studies, immersive or semi-immersive VR interventions demonstrated a measurable positive impact on participants’ emotional regulation, childbirth-related self-efficacy, and perceived control during pregnancy and labor. Several studies explicitly reported reductions in anxiety, depressive symptoms, or FOC, particularly when VR was used to visualize the childbirth process or to simulate relaxation exercises [34-38,40-43,48-51]. Physiological parameters also improved in studies integrating biobehavioral monitoring: VR-based prenatal education combined with perineal protection lowered pain scores and postpartum bleeding [23]; VR-guided relaxation stabilized uterine activity in women with preterm labor [47]; and VR-assisted exercise programs showed no adverse cardiovascular effects [46]. Not all outcomes moved in the same direction: an RCT of a VR operating room walkthrough before an elective cesarean did not significantly reduce preoperative anxiety overall, although participants (and partners) reported feeling better prepared and no motion sickness was observed [45]. Overall, 9 [23,34,40-42,45-48] out of 11 [23,34,40-48] (81.8%) studies reported at least 1 beneficial psychological or physiological outcome, while 4 [23,34,40,47] of 11 (36.4%) [23,34,40-48] showed statistically significant improvement in at least 1 quantitative measure. These findings suggest that immersive experiences can promote emotional stability, physiological adaptation, and empowerment during pregnancy and childbirth.

- Enhanced self-efficacy, confidence, and perceived control among women during childbirth (9/11, 81.2%): immersive learning strengthened mothers’ confidence and perceived control during childbirth [23,34,40-42,45-47].
- Distraction and emotional relief before and during childbirth (3/11, 27.3%): VR provided psychological distraction and emotional relief, reducing anxiety and stress through calming, immersive environments [34,40,45].
- Impact on physical or physiological parameters (3/11, 27.3%): immersive exposure was associated with reductions in pain and no adverse cardiovascular effects [23,46,47].

#### Theme 2: Customization, Sense of Presence, and Realism as Engagement Drivers

This theme captures how realism, presence, and adaptive learning features influence user engagement and satisfaction. Engagement was strongly shaped by audiovisual fidelity, a convincing sense of presence, and the possibility to tailor content. Users frequently described “being there” (eg, inside the birthing room) as key to relevance and motivation, and they valued options to progress independently [41,42]. At the same time, design tensions emerged when simulations looked or felt “game-like,” creating unmet expectations for guidance, interactivity, or rewards; these microfrictions could chip away at immersion if not intentionally balanced [43,44].

Based on the contents, the following 3 subthemes emerged:

- Realism, immersion, and sense of presence (4/11, 36.4%): authentic audiovisual fidelity fostered emotional connection and meaningful engagement (eg, 360° hospital tours and realistic birthing scenarios) [41-44].
- Customization and personalization (2/11, 18.2%): users valued flexibility and the ability to tailor learning pace and content to personal needs [43,44].
- Participant satisfaction (4/11, 36.4%): overall satisfaction with VR experiences was high compared with traditional courses [40,42,46,47].

#### Theme 3: Technical and Usability Considerations

This theme synthesizes participants’ feedback on the accessibility, usability, and technical limitations of immersive systems. Most users found VR interventions engaging, although minor barriers related to device comfort, navigation, or unfamiliarity with the technology were reported [44,46]. Feasibility and acceptability were generally good, with some device-related or interface hurdles. Multidevice access (HMD, computer, tablet, and smartphone) improved reach and scheduling flexibility, and HMDs often offered the best usability and presence [41,42].

The following subthemes are highlighted:

- Device flexibility and accessibility (5/11, 45.5%): VR allowed participation independent of place or schedule [34,40-42,44].
- Usability (ease of learning; 2/11, 18.2%): overall good usability, particularly with the headsets [41,42].
- Safety and tolerability: no serious adverse events reported (5/11, 45.5%) [23,34,40-42].
- Technological barriers (5/11, 45.5%): users occasionally experienced discomfort or interface issues [40-44].

#### Theme 4: VR as an Educational and Motivational Tool

This theme relates to cognitive learning, behavioral readiness, and social engagement facilitated by immersive tools. Most studies reported gains in knowledge, preparedness, and motivation. VR hospital tours about childbirth modules improved learning outcomes and provided concrete, realistic exemplars; VR-based programs also strengthened self-management and pregnancy care behaviors in higher-risk settings (eg, [42,47]). Immersive scenarios around breastfeeding surfaced lived challenges and promoted reflection and dialogue, complementing traditional didactics by addressing expectations and social or contextual barriers rarely covered in standard courses [43,44]. Preoperative VR for cesarean enhanced perceived preparedness in most women and their partners, highlighting motivational and informational value even when affective endpoints do not shift [45].

- Knowledge acquisition and retention (8/11, 72.7%): participants demonstrated improved understanding and recall of prenatal concepts [23,40-44,46,47].
- Ability to identify signs or symptoms of childbirth (5/11, 45.5%): VR improved recognition of early labor indicators and features related to breastfeeding experience [41,43,44,46,47].
- Coresponsibility and partner involvement (3/11, 27.3%): immersive education encouraged shared learning between partners [43-45].
- Recommendation to future parents (2/11, 18.2%): participants widely endorsed VR courses for peers [42,46].

#### Theme 5: Limitations

This final theme reflects methodological constraints and research priorities. Methodological constraints and implementation gaps temper generalizability. Several studies used small samples, single-group designs, or short follow-ups, and psychological outcomes were measured with heterogeneous tools. Selection biases and context-specific prototypes (eg, single-site pilots) were common, and partner or diverse-population representation was limited [40-43]. The main limits identified are the following:

1. Improving sample size (7/11, 63.6%) [34,40-42,45-47].
2. Lack of control group (3/11, 27.3%) [34,40,47].
3. Missing information (4/11, 36.4%): some studies pointed out the limited standardization especially about the measures used to assess psychological variables [23,40,42,45].
4. Selection biases (4/11, 36.4%) [34,41,45,46]: several studies point to the difficulty of accessing large and diverse samples, often having to resort to convenience sampling.
5. Confounding effect related to added intervention (VR and perineal protection [23]).
6. Inclusivity and contextual adaptation (4/11, 36.4%) [42-44,50].
7. Learning curve and technical literacy (4/11, 36.4%): limited familiarity with VR required orientation or staff support [42,43,46,47].
8. Lack of contextual and interactive cues and balanced gamification [42].
9. Lack of customization [34,44].
10. Lack of haptic feedback: the lack of perception of any type of pressure or vibration by participants may reduce their engagement with this technology [44].

### Synthesis of Thematic Findings

Overall, from the thematic analysis, it emerges that immersive interventions primarily enhance emotional regulation, self-efficacy, and knowledge retention, reported in 70%‐80% of the included studies. Engagement is strongly linked to realism, presence, and personalization, while usability and equity remain key implementation challenges (eg, [23,34,40,47]).

Despite small samples and methodological variability, the findings collectively portray VR as a promising, user-accepted innovation for prenatal and childbirth education. The 5 themes identified were closely interrelated rather than independent. Realism and sense of presence appeared to be a central mechanism linking the technological features of immersive scenarios with users’ psychological and educational outcomes. High levels of realism and immersion fostered emotional engagement and a stronger sense of preparedness, directly reinforcing confidence and learning outcomes. However, when the simulated experience became overly realistic or emotionally intense, participants occasionally reported discomfort, thereby connecting this theme to usability and instructional barriers. The need for structured guidance and emotional containment emerged as a mediating factor that shaped whether realism translated into positive or overwhelming experiences [41,44]. Similarly, the educational and motivational value of interventions was strongly influenced by the balance between realism and usability: participants were most motivated and confident when the immersive environment was both intuitive and emotionally manageable [42,43,47]. Finally, methodological and inclusivity limitations, such as small samples, short-term follow-up, and the underrepresentation of partners, acted as contextual moderators that constrained the generalizability of these interrelations [40,44,45]. Taken together, these interconnections suggest that the effectiveness of immersive scenarios in prenatal and childbirth education could be related to achieving equilibrium between immersion, usability, and emotional safety, ensuring that technological engagement consistently translates into meaningful learning and psychological benefit.

## Discussion

### Summary of Evidence

This scoping review underscores the growing importance of XR technologies in prenatal education, highlighting their diverse applications and potential benefits for expectant parents. Across heterogeneous designs and targets, XR (predominantly head-mounted VR and 360° video) emerged as a context-enabling technology that shapes expectations, self-efficacy, and preparedness.

The most consistently discussed mechanisms, warranting further investigation, were (1) presence and realism that situate users in concrete care pathways or lived scenarios (eg, birthing rooms and breastfeeding in public), aligning expectations with reality; (2) scaffolding (briefing, in-experience cues, and debriefing) to convert immersion into learning rather than overload; and (3) personalization and customization of pace, content, and intensity to match emotional readiness. These points recur in evaluations of childbirth education modules and breastfeeding simulations, which emphasize that simulations work best when they surface real-world complexity while remaining emotionally manageable [41-44]. Several included studies did not offer comprehensive VR-based childbirth training. Some interventions primarily targeted mental health promotion or general antenatal information, with childbirth skills training still provided via standard modalities; as a result, the evidence base appears stronger for preparedness and emotional regulation than for hands-on childbirth skills acquisition [34,40].

Not all affective end points shift uniformly. In the precesarean study using a 360° operating room walkthrough, overall preoperative anxiety did not significantly decline; however, participants felt better prepared and motion sickness was negligible, suggesting that VR may be more valuable for expectation setting and preparedness in this context [45]. By contrast, programs targeting mental health promotion during pregnancy reported reductions in depressive and anxiety symptoms and gains in quality of life, with stronger effects in higher-severity subgroups, consistent with VR acting as a precision amplifier when distress is greater [34,40]. This pattern aligns with broader VR evidence showing clinically meaningful anxiolytic and analgesic effects across nonobstetric contexts and robust efficacy for anxiety disorders in randomized trials [53-55].

In a recent noninferiority randomized trial evaluating an MR adjunct to standard prenatal counseling, there were no between-group differences in maternal self-efficacy or satisfaction at 1 week postpartum; however, exclusive breastfeeding reached 93.1% in both arms, indicating high baseline supports and potential ceiling effects [48].

Where VR was embedded alongside biobehavioral cointerventions (eg, perineal-protection techniques taught within a VR-supported prenatal program), studies discussed pragmatic benefits (eg, lower pain) while cautioning that future trials must disentangle the VR effect from the added clinical component [23]. From a design perspective, breastfeeding simulations repeatedly highlight a productive tension: when experiences feel game-like, users expect goals, feedback, and progression; if those are absent, microfrictions can erode engagement. Authors, therefore, advocate measured gamification (clear objectives and contextual feedback) and structured debriefs that channel emotional impact into reflection and action, principles that generalize to other perinatal VR modules [43,44]. Finally, implementation work converges on a practical message: HMDs maximize presence and usability for key sessions, but multidevice access (HMD plus PC or tablet or phone) broadens reach and equity for rehearsal and partner involvement. Brief sessions, seated posture, orientation, and clinician facilitation improve tolerance and learning transfer [41-44,47].

Findings converge with nonobstetric meta-analyses reporting consistent VR-driven reductions in pain and anxiety, and with clinical education studies where brief, expectation-aligned VR modules improve understanding when embedded in routine workflows [53-56]. In obstetrics, pooled analyses suggest state anxiety reductions during routine procedures without consistent maternal-fetal safety signals, albeit with reporting heterogeneity and short follow-up [24,57,58]. Overall, our results extend this literature by focusing on parent-facing prenatal education, emphasizing emotional preparedness, usability across digital literacy levels, and the value of facilitated debriefing, which are less central in clinician training research.

### Implications for Clinical Practice

The findings of this scoping review suggest several concrete ways in which XR can be incorporated into prenatal care pathways. For parents scheduled for elective cesarean delivery, VR appears particularly useful as an informational and pathway-familiarization tool. Short, guided 360° tours of the operating theater and perioperative environment can strengthen perceived preparedness, even when mean anxiety scores do not change, and may be especially valuable for those with a history of emergency cesarean or heightened fear of surgery. Wherever possible, partners should be included so that both members of the dyad share a realistic understanding of the procedure and the perioperative setting [45].

Immersive programs that primarily target antenatal mental health, combining positive affect induction, psychoeducation, and relaxation or mindfulness elements, have shown reductions in depressive and anxiety symptoms and improvements in quality of life, with larger effects among higher-severity subgroups [34,41,42,46,47]. Clinically, this pattern supports the use of VR as an adjunctive component within stepped care models rather than as a stand-alone treatment. Brief, structured sessions can be embedded in routine antenatal visits to support emotional regulation, while more intensive psychotherapeutic strategies are likely to remain necessary when the aim is to modify stress appraisal and coping, which changed less consistently in the included trials [34,41,42,46,47]. Converging evidence from nonobstetric meta-analyses supports VR’s adjunct role in reducing state anxiety in routine care pathways [53-55].

Within childbirth education and early parenting programs, headset-based, scenario-driven modules can be reserved for key sessions that introduce labor environments, decision points, and immediate postnatal routines, whereas remote 360° or video content can serve as refreshers to consolidate learning and allow parents to revisit material together at home [43-45].

For breastfeeding preparation, lived experience simulations that depict common technical and social challenges, such as latching difficulties or feeding in public, can help parents anticipate and normalize difficulties. Their impact appears greatest when VR sessions are framed and debriefed by midwives or lactation consultants, turning emotionally salient experiences into concrete action plans. Evidence from neonatal intensive care settings further suggests that VR may support lactation among mothers of preterm infants by reducing anxiety and enhancing expressed milk volume, although effects on self-efficacy and satisfaction depend on the quality of underlying counseling [43,44,48,59].

Across all applications, implementation choices shape both effectiveness and safety. The studies included in this review indicate that keeping sessions brief (around 10‐15 min), using a seated posture, and allowing user-controlled pacing minimizes cybersickness and fatigue; no serious adverse events were reported, and obstetric meta-analyses likewise describe stable maternal and fetal parameters during short, guided uses [45,60].

When experiences adopt game-like conventions, it is important to make learning goals explicit and to provide light-touch feedback (eg, progress cues or hints), so that playfulness sustains engagement without trivializing care content [43,44]. Coviewing and partner involvement generally improve dyadic preparedness and adherence [45], and hybrid delivery models, combining clinic-based HMDs for high-presence moments with multidevice access (HMD, tablet, or smartphone) for rehearsal at home, may offer a pragmatic balance between fidelity, reach, and equity in diverse populations [41-44,47,61-64].

### Implications for Future Research

Future research on XR-based prenatal education would benefit from greater methodological convergence. Across the included studies, outcome measures for anxiety, FOC, self-efficacy, and preparedness were highly fragmented, and reporting of presence, realism, and debriefing formats was inconsistent. Developing core outcome sets and agreed-upon reporting standards for VR “dose” (eg, frequency, duration, and hardware characteristics) and facilitation strategies would enhance comparability and support robust meta-analysis. Safety reporting should routinely include maternal hemodynamics, fetal heart rate patterns, and neonatal indicators, in line with emerging obstetric meta-analyses on intrapartum VR [24,40-44,47,50,57].

Several trials suggest that immersive programs may be particularly helpful for individuals with higher baseline distress, such as those with elevated depression or anxiety scores, or pronounced childbirth fear [34,41,42,46,47]. This pattern points to the need for studies that are prospectively powered for subgroup analyses, include prespecified interaction tests, and, where feasible, use adaptive designs to identify who benefits most and at what time point in pregnancy. In addition, family-centered outcomes, such as partner preparedness, dyadic coping, and coparenting adjustment, remain undermeasured despite growing evidence on paternal mental health and engagement. Future trials should therefore integrate these end points and draw on paternal-engagement frameworks when designing XR content [61-64].

Many of the interventions identified in the current review combined VR with additional components, including perineal protection techniques, structured counseling, or hands-on practice with task trainers, which makes it difficult to isolate the specific contribution of immersion. Future work should use active comparators or factorial designs to disentangle VR effects from cointerventions and to quantify how presence, guidance, and repetition relate to psychological and clinical outcomes [23]. Insights from rehabilitation science, where VR has been used extensively to structure motor learning schedules, could help specify dose-response relationships and practice parameters for perinatal education [65]. In parallel, design lessons emerging from breastfeeding simulations and co-design prototypes, such as embracing uncertainty while offering context-relevant feedback and enabling customization of family composition and environments, should be translated into testable design requirements and evaluated prospectively, even when initial prototypes fall outside strict effectiveness trial criteria [43,44]. Finally, implementation-focused and real-world research is needed to understand how immersive prenatal education can be scaled and sustained. Multicenter pragmatic trials should track uptake, completion rates, costs, and equity impacts, including representation of underserved groups and partner participation [41-44,47]. Given that early studies often restricted the use of headsets to clinic settings and resorted to nonimmersive devices for remote delivery, future work should evaluate models that provide loaner HMDs and home-based protocols with remote safety monitoring, comparing them with non-HMD approaches in terms of adherence, safety, user experience, and outcomes. Such implementation studies can inform guidelines for integrating XR into hybrid antenatal care pathways while safeguarding accessibility, inclusivity, and safety.

### Limitations

Although our search spanned XR broadly, included studies were almost exclusively VR or 360°, with 1 MR trial, underscoring a modality gap [48]. Small samples, single-site prototypes, short follow-up, and heterogeneous measures limit generalizability and synthesis; some HCI-led prototypes caution that game-like framing can create normative expectations unless customization is provided [34-38,40-47,49-51]. Acceptability and safety were high overall, yet digital literacy and brief orientation remain prerequisites; where VR is embedded in multicomponent care, physiologic and clinical end points appear promising but attribution to VR alone is uncertain [23]. In obstetrics, evidence suggests anxiety reductions without consistent safety concerns, but reporting remains variable [24,57,58]. Finally, RCT work in breastfeeding points to targeted experiential benefits warranting replication and linkage to clinical end points [66].

### Conclusions

For perinatal care, VR is most effective when high presence is matched with structured guidance and personalization, turning immersive exposure into meaningful preparedness and emotional regulation. Clinically, this translates into targeted use (eg, elective cesarean preparation and higher-severity mental health profiles) and hybrid delivery that balances fidelity with reach. For the field to mature, we need standardized outcomes, subgroup-powered pragmatic trials, and designs that separate VR’s contribution from cointerventions while operationalizing HCI insights (uncertainty, feedback, and customization) as testable requirements. Integrating insights from broader VR meta-analyses and adjacent pediatric and rehabilitation domains strengthens the rationale for perinatal VR, while pointing to concrete design and implementation levers, partner inclusion, at-home HMD access, and safety-standardized reporting, that can accelerate translation into routine care.

## Supplementary material

10.2196/83621 Multimedia Appendix 1 Search Strategy.

10.2196/83621 Checklist 1 PRISMA-ScR checklist.

## Abbreviations

ADDIE: Analyze, Design, Develop, Implement, and Evaluate
AR: augmented reality
CINAHL: Cumulative Index to Nursing and Allied Health Literature
EPDS: Edinburgh Postnatal Depression Scale
FOC: fear of childbirth
GAD-7: Generalized Anxiety Disorder-7
HCI: human-computer interaction
HMD: head-mounted display
IMCHB: Interaction Model of Client Health Behavior
MR: mixed reality
OSF: Open Science Framework
PHQ-9: Patient Health Questionnaire-9
PRISMA: Preferred Reporting Items for Systematic Reviews and Meta-Analyses
PRISMA-ScR: Preferred Reporting Items for Systematic Reviews and Meta-Analyses extension for Scoping Reviews
PSS: Perceived Stress Scale
RCT: randomized controlled trial
RQ: research question
STAI: State-Trait Anxiety Inventory
VR: virtual reality
W-DEQ: Wijma Delivery Expectancy/Experience Questionnaire
WHO: World Health Organization
WHOQOL-BREF: World Health Organization Quality of Life–BREF
WoS: Web of Science
XR: extended reality
