# Supplementary material for: Virtual, Augmented, Mixed, and Immersive Technologies for Prenatal and Childbirth Education: Scoping Review
Source: JMIR Pediatr Parent. 2026 May 1;9:e83621. doi: 10.2196/83621 (PMC13133985; doi:10.2196/83621)
Supplement: Multimedia Appendix 1 [file pediatrics-v9-e83621-s001.docx]

**Multimedia Appendix 1 – Search Strategy**

This supplementary file details the database-specific search strategies employed in the scoping review. Searches were conducted up to the 16 October 2025. Boolean operators were used where appropriate to enhance retrieval. Minor syntax variations were introduced to accommodate database-specific query requirements.

**Scopus**

("virtual reality" OR "VR" OR "augmented reality" OR "mixed reality" OR "extended reality" OR "immersive technology") AND ("prenatal education" OR "antenatal education" OR "childbirth education" OR "prenatal classes" OR "parenting classes" OR "expectant parent education" OR "childbirth training") AND ("pregnant women" OR "expectant parents" OR "parents" OR "midwife" OR "healthcare provider" OR "childbirth educator" OR "parent educator" OR "health professional" OR "perinatal educator") AND NOT ("medical student" OR "nursing student" OR "midwifery student" OR "clinician training" OR "health professional training" OR "simulation for healthcare professionals")

**Web of Science**

TS=("virtual reality" OR VR OR "augmented reality" OR "mixed reality" OR "extended reality" OR "immersive technology") AND TS=("prenatal education" OR "antenatal education" OR "childbirth education" OR "prenatal classes" OR "parenting classes" OR "expectant parent education" OR "childbirth training") AND TS=("pregnant women" OR "expectant parents" OR "parents" OR "midwife" OR "healthcare provider" OR "childbirth educator" OR "parent educator" OR "health professional" OR "perinatal educator") AND NOT TS=("medical student" OR "nursing student" OR "midwifery student" OR "clinician training" OR "health professional training" OR "simulation for healthcare professionals")

**IEEE Xplore**

("virtual reality" OR "VR" OR "augmented reality" OR "mixed reality" OR "extended reality" OR "immersive technology") AND ("prenatal education" OR "antenatal education" OR "childbirth education" OR "prenatal classes" OR "parenting classes" OR "expectant parent education" OR "childbirth training") AND ("pregnant women" OR "expectant parents" OR "parents" OR "midwife" OR "healthcare provider" OR "childbirth educator" OR "parent educator" OR "health professional" OR "perinatal educator") NOT ("medical student" OR "nursing student" OR "midwifery student" OR "clinician training" OR "health professional training" OR "simulation for healthcare professionals")

**PUBMED**

("virtual reality"[Title/Abstract] OR "VR"[Title/Abstract] OR "augmented reality"[Title/Abstract] OR "mixed reality"[Title/Abstract] OR "extended reality"[Title/Abstract] OR "immersive technology"[Title/Abstract] OR "Virtual Reality"[MeSH]) AND ("prenatal education"[Title/Abstract] OR "childbirth education"[Title/Abstract] OR "antenatal education"[Title/Abstract] OR "prenatal classes"[Title/Abstract] OR "parenting classes"[Title/Abstract] OR "expectant parent education"[Title/Abstract] OR "childbirth training"[Title/Abstract] OR "Prenatal Education"[MeSH] OR "Parenting Education"[MeSH] OR "Education, Nonprofessional"[MeSH]) AND ("pregnant women"[Title/Abstract] OR "expectant parents"[Title/Abstract] OR "parents"[Title/Abstract] OR "midwife"[Title/Abstract] OR "healthcare provider"[Title/Abstract] OR "childbirth educator"[Title/Abstract] OR "parent educator"[Title/Abstract] OR "health professional"[Title/Abstract] OR "perinatal educator"[Title/Abstract]) NOT ("medical student"[Title/Abstract] OR "nursing student"[Title/Abstract] OR "midwifery student"[Title/Abstract] OR "clinician training"[Title/Abstract] OR "health professional training"[Title/Abstract] OR "simulation for healthcare professionals"[Title/Abstract])

**CINAHL Ultimate / EBSCOhost**

("virtual reality" OR "VR" OR "augmented reality" OR "mixed reality" OR "extended reality" OR "immersive technology") AND ("prenatal education" OR "antenatal education" OR "childbirth education" OR "prenatal classes" OR "parenting classes" OR "expectant parent education" OR "childbirth training") AND ("pregnant women" OR "expectant parents" OR "parents" OR "midwife" OR "healthcare provider" OR "childbirth educator" OR "parent educator" OR "health professional" OR "perinatal educator") NOT ("medical student" OR "nursing student" OR "midwifery student" OR "clinician training" OR "health professional training" OR "simulation for healthcare professionals")

**APA PsychINFO** / **APA PsychArticles**

("virtual reality" OR "VR" OR "augmented reality" OR "mixed reality" OR "extended reality" OR "immersive technology") AND ("prenatal education" OR "antenatal education" OR "childbirth education" OR "prenatal classes" OR "parenting classes" OR "expectant parent education" OR "childbirth training") AND ("pregnant women" OR "expectant parents" OR "parents" OR "midwife" OR "healthcare provider" OR "childbirth educator" OR "parent educator" OR "health professional" OR "perinatal educator") NOT ("medical student" OR "nursing student" OR "midwifery student" OR "clinician training" OR "health professional training" OR "simulation for healthcare professionals")
